# RIM-Binding Protein 2 Organizes Ca^2+^ Channel Topography and Regulates Release Probability and Vesicle Replenishment at a Fast Central Synapse

**DOI:** 10.1523/JNEUROSCI.0586-21.2021

**Published:** 2021-09-15

**Authors:** Tanvi Butola, Theocharis Alvanos, Anika Hintze, Peter Koppensteiner, David Kleindienst, Ryuichi Shigemoto, Carolin Wichmann, Tobias Moser

**Affiliations:** ^1^Institute for Auditory Neuroscience and InnerEarLab, University Medical Center Göttingen, Göttingen 37075, Germany; ^2^Synaptic Nanophysiology Group, Max Planck Institute for Biophysical Chemistry, 37077 Göttingen, Germany; ^3^International Max Planck Research School for Neurosciences and Göttingen Graduate Center for Neurosciences, Biophysics, and Molecular Biosciences (GGNB), University of Göttingen, 37075 Göttingen, Germany; ^4^Collaborative Research Center 1286, University of Göttingen, 37075 Göttingen, Germany; ^5^Molecular Architecture of Synapses Group, Institute for Auditory Neuroscience and Center for Biostructural Imaging of Neurodegeneration, 37075 Göttingen, Germany; ^6^Institute of Science and Technology Austria (IST Austria), 3400 Klosterneuburg, Austria; ^7^Cluster of Excellence “Multiscale Bioimaging: from molecular machines to networks of excitable cells,” University of Göttingen, Göttingen 37075, Germany

**Keywords:** active zone topography, calyceal synapses, Ca_V_2.1 control of transmitter release, endbulb of Held, RIM-binding protein 2, short-term plasticity

## Abstract

Rab-interacting molecule (RIM)-binding protein 2 (BP2) is a multidomain protein of the presynaptic active zone (AZ). By binding to RIM, bassoon (Bsn), and voltage-gated Ca^2+^ channels (Ca_V_), it is considered to be a central organizer of the topography of Ca_V_ and release sites of synaptic vesicles (SVs) at the AZ. Here, we used RIM-BP2 knock-out (KO) mice and their wild-type (WT) littermates of either sex to investigate the role of RIM-BP2 at the endbulb of Held synapse of auditory nerve fibers (ANFs) with bushy cells (BCs) of the cochlear nucleus, a fast relay of the auditory pathway with high release probability. Disruption of RIM-BP2 lowered release probability altering short-term plasticity and reduced evoked EPSCs. Analysis of SV pool dynamics during high-frequency train stimulation indicated a reduction of SVs with high release probability but an overall normal size of the readily releasable SV pool (RRP). The Ca^2+^-dependent fast component of SV replenishment after RRP depletion was slowed. Ultrastructural analysis by superresolution light and electron microscopy revealed an impaired topography of presynaptic Ca_V_ and a reduction of docked and membrane-proximal SVs at the AZ. We conclude that RIM-BP2 organizes the topography of Ca_V_, and promotes SV tethering and docking. This way RIM-BP2 is critical for establishing a high initial release probability as required to reliably signal sound onset information that we found to be degraded in BCs of RIM-BP2-deficient mice *in vivo*.

**SIGNIFICANCE STATEMENT** Rab-interacting molecule (RIM)-binding proteins (BPs) are key organizers of the active zone (AZ). Using a multidisciplinary approach to the calyceal endbulb of Held synapse that transmits auditory information at rates of up to hundreds of Hertz with submillisecond precision we demonstrate a requirement for RIM-BP2 for normal auditory signaling. Endbulb synapses lacking RIM-BP2 show a reduced release probability despite normal whole-terminal Ca^2+^ influx and abundance of the key priming protein Munc13-1, a reduced rate of SV replenishment, as well as an altered topography of voltage-gated (Ca_V_)2.1 Ca^2+^ channels, and fewer docked and membrane proximal synaptic vesicles (SVs). This hampers transmission of sound onset information likely affecting downstream neural computations such as of sound localization.

## Introduction

Active zones (AZs) are specialized regions at the presynaptic terminals where neurotransmitter release occurs. AZs employ a sophisticated machinery to enable ultrafast coupling of the incoming action potential to the release of transmitter via Ca^2+^-triggered synaptic vesicle (SV) fusion. Voltage-gated Ca^2+^ channels (Ca_V_) and SV release sites represent the core machinery, and their relative topography at the AZ co-determines the release probability (for recent review, see Kaeser and Regehr, 2014; Schneggenburger and Rosenmund, 2015; Walter et al., 2018; Dittman and Ryan, 2019). The function and abundance of Ca_V_ (for recent review, see Pangrsic et al., 2018; Dolphin and Lee, 2020) is positively regulated by auxiliary subunits and multidomain proteins of the AZ such as Rab-interacting molecule (RIM)-binding protein (BP), RIM, piccolo, bassoon (Bsn), CAST, and ELKS.

Several of these proteins promote the clustering of Ca^2+^ channels at the AZ and/or their interaction with the SV release sites (Gundelfinger and Fejtova, 2012; Südhof, 2012; Moser et al., 2020). RIM and RIM-BPs, in particular, have been considered as molecular linkers of Ca_V_ and vesicular release sites. Direct and indirect interactions of RIMs with Ca_V_ are well established (Kiyonaka et al., 2007; Gebhart et al., 2010; Kaeser et al., 2011; Picher et al., 2017). Proline-rich sequences of RIMs have been shown to bind the SH3 domains of RIM-BP that directly interacts with Ca_V_ (Hibino et al., 2002; Kaeser et al., 2011). Deletion of *Drosophila* RIM-BP disrupted Ca_V_ clustering at the AZs of larval neuromuscular junctions consequently impairing their functional coupling to SV release: altered short-term plasticity demonstrating a reduced release probability (Liu et al., 2011). Of the three mammalian RIM-BP isoforms, RIM-BP1 and RIM-BP2 are neuron specific, whereby RIM-BP2 seems to be the isoform that is most relevant for synaptic transmission (Acuna et al., 2015; Grauel et al., 2016; Krinner et al., 2021). Disruption of RIM-BP1 and RIM-BP2 did not alter the Ca^2+^ current at the calyx of Held synapse (Acuna et al., 2015), while lack of RIM-BP2 (Krinner et al., 2017; Luo et al., 2017) and of RIM-BP1 (Luo et al., 2017) reduced the Ca_V_ number at ribbon-type AZs. An alteration of the Ca_V_ topography at the AZs has been reported based on superresolution immunofluorescence microscopy (Grauel et al., 2016; Krinner et al., 2017).

A loosening of the otherwise tight spatial relationship of Ca_V_ and SV release sites was indicated by the reduced release probability and a greater sensitivity to the intracellular presence of the “slow” Ca^2+^ chelator EGTA in the absence of RIM-BP(1)/2 (Acuna et al., 2015; Grauel et al., 2016; Luo et al., 2017). A similar conclusion was reached in sensory hair cells where, unlike in CNS synapses, SV replenishment was impaired in the absence of RIM-BP2 (Krinner et al., 2017). However, in contrast to the dramatic impairment of synaptic transmission at RIM-BP-deficient neuromuscular junctions in *Drosophila* (Liu et al., 2011), transmission was affected more subtly by RIM-BP deletion at mammalian synapses (Acuna et al., 2015; Grauel et al., 2016; Krinner et al., 2017; Luo et al., 2017). This might relate to partial compensation by other candidate linkers of Ca_V_ and SV release sites such as long RIM isoforms (Acuna et al., 2016). Recently, two alternative actions of RIM-BPs were indicated based on molecular perturbations studies in hippocampal neurons: (1) binding to Ca_V_ enabling their tight coupling to SVs or (2) promoting SV priming by interaction with Munc13-1 (Brockmann et al., 2020). Here, we aimed to further characterize the function of RIM-BP2 at the endbulb of Held synapse, the first central auditory synapse (von Gersdorff and Borst, 2002; Yu and Goodrich, 2014), that employs high vesicular release probability at its >100 AZs for reliable and temporally precise signal transmission from auditory nerve fibers (ANFs) to bushy cells (BCs) at frequencies of hundreds of Hertz (Trussell, 1999; Wang et al., 2011). We combined electrophysiological analysis with studies of the molecular composition and ultrastructure of the AZ in RIM-BP2-deficient endbulbs.

## Materials and Methods

### Animals

The constitutive RIM-BP2 knock-out (RIM-BP2 KO) mice were described previously (Grauel et al., 2016) and kindly provided to us by Dr. Christian Rosenmund, Dr. Katharina Grauel, and Dr. Stephan Sigrist. RIM-BP2 KO mice and their wild-type littermates (RIM-BP2 WT) of either sex were studied after the onset of hearing (postnatal day 13). For specific animal ages, please refer to the relevant Materials and Methods sections. All experiments were performed in compliance with the guidelines of the German animal welfare act and were approved by the board for animal welfare of the University Medical Center Göttingen and the animal welfare office of the state of Lower Saxony.

### Slice electrophysiology

#### Slice preparation

Acute parasagittal slices (150 µm) from the anteroventral cochlear nucleus (aVCN) were obtained as described previously (Mendoza Schulz et al., 2014). Briefly, after killing by decapitation, brains were dissected out and quickly immersed in ice-cold low Na^+^ and low Ca^2+^ cutting solution containing the following: 50 mm NaCl, 26 mm NaHCO_3_, 120 mm sucrose, 1.25 mm NaH_2_PO_4_.H_2_O, 2.5 mm KCl, 20 mm glucose, 0.2 mm CaCl_2_, 6 mm MgCl_2_, 0.7 mm Na L-ascorbate, 2 mm Na pyruvate, 3 mm myo-inositol, and 3 mm Na L-lactate with pH adjusted to 7.4 and osmolarity of around 310 mOsm/l. After removal of the meninges from the ventral face of the brainstem, the two hemispheres were separated by a midsagittal cut, and the forebrain was removed at the pons-midbrain junction. The brain blocks containing brain stem and cerebellum were then glued (cyanoacrylate glue; Loctite 401, Henkel) to the stage of a VT 1200S vibratome (Leica Microsystems) such that the medial side was glued on, the ventral side was facing the blade and the lateral side was facing upwards, submerged in ice-cold cutting solution. For sectioning, the blade was positioned at the height of cerebellar flocculus and sections were cut at a blade feed rate of 0.02 mm/s with an amplitude of 1.50 mm. Slices were incubated for 30 min in artificial CSF (aCSF) maintained at 35°C, and then kept at room temperature (22–24°C) until recording. Composition of aCSF was identical to the cutting solution except 125 mm NaCl, 13 mm glucose, 2 mm CaCl_2_, and 1 mm MgCl_2_. The pH of the solution was adjusted to 7.4 and osmolarity was around 310 mOsm/l. All solutions were continuously aerated with carbogen (95% O_2_, 5% CO_2_). For presynaptic endbulb recordings, coronal sections were made instead of parasagittal as described above. The only difference was that after the forebrain was removed at the pons-midbrain junction, the dissected brain was not cut midsagitally and the brain block containing the brain stem and the cerebellum was then glued to the vibratome stage with the caudal aspect facing upwards and the ventral side toward the blade.

#### Electrophysiology

Patch-clamp recordings were made from BCs of aVCN using EPC10 USB Patch clamp amplifier controlled by the Patchmaster software (HEKA Elektronik). Sampling interval and filter settings were 25 µs and 7.3 kHz, respectively. Cells were visualized by differential interference contrast (DIC) microscopy through a 40× water-immersion objective (NA 0.8; Zeiss) using an Axioscope 2 FS plus microscope (Zeiss). All experiments were conducted at a temperature of 33–35°C, maintained by constant superfusion (flow rate 3–4 ml/min) of aCSF, heated by an inline solution heater (SH-27B with TC-324B controller; Warner Instruments) and monitored by a thermistor placed between the inflow site and the slice, in the recording chamber.

Patch pipettes were pulled with P-87 micropipette puller (Sutter Instruments Co) from borosilicate glass capillaries with filament (GB150F, 0.86 × 1.50 × 80 mm; Science Products). Open tip pipette resistance was 1.5–3 MΩ when filled with intracellular solution containing the following: 115 mm K-gluconate, 10 mm HEPES, 8 mm EGTA, 10 mm Na_2_phosphocreatine, 4 mm ATP-Mg, 0.3 mm GTP-Na, 4.5 mm MgCl_2_, 10 mm NaCl, and 1 mm *N*-(2,6-dimethylphenyl carbamoylmethyl) triethylammonium chloride (QX-314; Alomone Labs) to block sodium channels, with a pH of 7.35 and an osmolarity of 300 mOsm/l. Additionally, 1 mm of fluorescent dye Alexa Fluor 488 (Invitrogen) was added to the recording pipette and cell structure was examined during experiments using a HXP 120 mercury lamp, with FITC filter (Semrock hardcoat). BCs were identified by the faster kinetics of their postsynaptic currents (Isaacson and Walmsley, 1995) and their characteristic short-term plasticity (Chanda and Xu-Friedman, 2010) and selection confirmed by their spherical in appearance with one primary dendrite terminating in a dense bush-like dendritic tree (Wu and Oertel, 1984), Cells were voltage-clamped at a holding potential of –70 mV, after correction for a liquid junction potential of 12 mV. Mean series resistance was around 5 MΩ and was compensated up to 70% with a 10-µs lag. Presynaptic ANF were minimally stimulated with a monopolar electrode in a patch pipette filled with aCSF, placed at a distance of at least three cell diameters from the cell being recorded. Stimulating currents of 10–20 µA were delivered through a stimulus isolator (A360 World Precision Instruments). For the main set of recordings, bath solution (aCSF) was supplemented with the following: 1 mm kynurenic acid sodium salt (Abcam Biochemicals), a low-affinity AMPAR antagonist, to prevent receptor saturation/desensitization, 100 µm cyclothiazide (CTZ) to prevent AMPAR desensitization, 10 µm bicuculline methchloride, a GABA_A_ receptor antagonist and 2 µm strychnine hydrochloride, a glycine receptor antagonist.

For patch-clamp experiments at the endbulbs of Held, patch-pipettes were coated with either dental wax or Sylgard to minimize fast capacitive transients and stray capacitance during voltage-clamp experiments. Open tip pipette resistance was 4–5 MΩ with an intracellular solution containing the following: 130 mm Cs-methanesulfonate, 20 mm TEA-Cl, 10 mm HEPES, 0.5 mm EGTA, 5 mm Na_2_phosphocreatine, 4 mm ATP-Mg, and 0.3 mm GTP-Na, with pH adjusted to 7.3 with CsOH and an osmolarity of 320 mOsm/l. For anatomic confirmation 1 mm of fluorescent dye Alexa Fluor 488 (Invitrogen) was added to the recording pipette. The bath solution differed from the aCSF normally used in the following: 85 mm NaCl, 25 mm glucose, 2 mm CaCl_2_, and 1 mm MgCl_2_. The pH of the solution was adjusted to 7.4 and osmolarity was around 310 mOsm/l. Additionally, the bath solution was supplemented with 1 µm TTX, 1 mm 4-AP, and 40 mm TEA-Cl to suppress voltage-gated Na^+^ and K^+^ currents. Presynaptic terminals were voltage-clamped at a holding potential of –80 mV, a liquid junction potential of 3 mV was ignored. Series resistance was <30 MΩ and was compensated up to 50% with a 10-µs lag.

### Systems physiology: extracellular recordings from single BCs

Extracellular recordings from single units of BCs in the aVCN were performed as described before (Jing et al., 2013; Strenzke et al., 2016) in 9- to 10-week-old mice. After anesthetizing the mice with intraperitoneal injection of urethane (1.32 mg/kg), xylazine (5 mg/kg), and buprenorphine (0.1 mg/kg), a tracheostomy was performed, their cartilaginous ear canals were removed and then they were positioned in a custom-designed head-holder and stereotactic system. After partial removal of the occipital bone and cerebellum to expose the surface of the cochlear nucleus, a glass microelectrode was advanced through the anterior portion of the aVCN to avoid the ANFs and instead target the area with a higher fraction of spherical BCs. Acoustic stimulation was provided by an open field Avisoft ScanSpeak Ultrasonic Speaker (Avisoft Bioacoustics). “Putative” spherical BCs were identified and differentiated from other cell types in the cochlear nucleus by their characteristic “primary-like” peristimulus time histogram (PSTH; Taberner and Liberman, 2005), irregular firing pattern demonstrated by a ≥0.5 coefficient of variation (c.v.) of interspike intervals of adapted responses, and a first spike latency of ≤5 ms. BCs units were distinguished from ANF (also having primary-like PSTH) based on their stereotactic position (<1.1 mm below the surface of the cochlear nucleus). Recordings were performed using TDT system III hardware and an ELC-03XS amplifier (NPI Electronics).

### Immunohistochemistry and confocal imaging

Mice at postnatal days 20–24 were deeply and terminally anesthetized with xylazin (5 mg/kg) and ketamin (10 mg/ml) in 0.9% saline and then transcardially perfused with 2% freshly prepared ice-cold paraformaldehyde with pH adjusted to 7.4. The fixed brain was then removed and brainstem was dissected with a coronal cut few millimeters nasal to the junction between occipital cortex and cerebellum. The brain block was washed overnight in 30% sucrose solution in PBS. For sectioning, the brain block was embedded in Tissue Tek Cryomatrix (Thermo Fisher Scientific) and then fixed on the stage of the cryostat (Figocut E cryotome, Reichert-Jung) such that the caudal aspect was facing upwards and the dorsal side was toward the blade. Advancing from caudal to nasal, 30-µm coronal sections were cut (chamber temperature: −20°C, object temperature: −22°C) and discarded until the appearance of the seventh cranial nerve. Subsequent sections containing aVCN were collected onto electrostatically charged microscope slides (SuperFrost Plus, Thermo Fisher Scientific). For parallel processing, one slice of each genotype was collected per slide. Thereafter, the slices were washed for 10 min in PBS and incubated in goat serum dilution buffer [GSDB; 16% normal goat serum, 450 mm NaCl, 0.3% Triton X-100, and 20 mm phosphate buffer (PB), pH 7.4] for 1 h, followed by incubation in primary antibodies diluted in GSDB, for 3 h, in a wet chamber at room temperature. After washing 2 × 10 min with wash buffer (450 mm NaCl, 0.3% Triton X-100, and 20 mm PB) and 2 × 10 min with PBS, the slices were incubated with secondary antibodies diluted in GSDB, for 1 h, in a light-protected wet chamber at room temperature. The slices were then washed 2 × 10 min with wash buffer, 2 × 10 min with PBS and 1 × 10 min in 5 mm PB, and finally mounted with a drop of fluorescence mounting medium based on Mowiol 4–88 (Carl Roth) and covered with a thin glass coverslip. The above-described perfusion fixation method was used to stain for RIM-BP2. The remaining immunofluorescence experiments (Munc13-1, RIM2, CAST, Bsn) were done on samples taken from RIM-BP2 KO mice aged between postnatal day (p)15 and p23 and WT littermates. After obtaining coronal sections of the unfixed mouse brain from the cryostat the slices were maintained frozen at −20°C until immersion fixation or fixed directly on ice in a solution of PBS containing 3% w/v heat depolymerized PFA (70°C) for 3 min. This alternative method of fixation showed robust labeling with less background than perfusion fixed samples labeled against the same markers. For the comparison of Ca_V_2.1 immunofluorescence levels in confocal microscopy and the stimulated emission depletion (STED) analysis of Ca_V_2.1 and Bsn clusters, we used very brief fixation with 3% PFA for <1 min (30–40 s) as Ca_V_2.1 labeling was impeded by stronger fixation. Thereafter, the blocking and immunolabeling protocols were followed exactly as described above for the perfusion fixed samples.

Primary antibodies used were: rabbit anti-RIM-BP2 (1:200), guinea pig anti-VGLUT1 (1:500), rabbit anti-VGLUT1 (1:1000), mouse anti-Gephyrin (1:500), mouse anti-Sap7f407 to Bsn (1:500; Abcam), guinea pig anti-Bsn (1:500), rabbit anti-Munc13-1 (1:200), rabbit anti-RIM1 (1:200), rabbit anti-RIM2 (1:200), rabbit anti-CAST (1:200), rabbit anti-P/Q Ca^2+^ channel (1:500), chicken anti-Homer1 (1:200). Unless stated otherwise, primary antibodies were purchased from Synaptic Systems. Secondary antibodies used were: Alexa Fluor 488-, Alexa Fluor 568-, and Alexa Fluor 647-labeled antibodies (1:200, Invitrogen), goat anti-guinea pig STAR580, and goat anti-rabbit STAR 635p (1:200, Abberior GmbH).

Confocal images were acquired using a laser-scanning confocal microscope (Leica TCS SP5; Leica Microsystems) equipped with 488-nm (Ar) and 561/633-nm (He-Ne) lasers and 63×/1.4 NA oil-immersion objective. STED and confocal images (Ca_V_2.1 and Bsn analyses) were acquired using a two-color STED microscope (Abberior Instruments) equipped with 561- and 640-nm excitation lasers, a 775-nm laser for STED (1.2 W) and a 100× oil immersion objective (1.4 NA, Olympus). Confocal *z*-stacks were processed with Imaris (Bit-plane) for spot detection, co-localization analysis and fluorescence intensity calculation using custom MATLAB scripts. STED images of Ca_V_2.1 and Bsn were analyzed using Igor Pro7 (Wavemetrics). Samples of both genotypes: RIM-BP2 WT and RIM-BP2 KO were processed and imaged in parallel, using same laser power, gain, and microscope settings.

### SDS–freeze-fracture replica immunolabeling (SDS-FRIL)

Mice at postnatal days 20–24 were deeply and terminally anesthetized with xylazin (5 mg/kg) and ketamin (10 mg/ml) in 0.9% saline, and perfused transcardially with ice-cold PBS followed by perfusion with freshly-prepared 2% PFA with 15% saturated picric acid solution in 0.1 m PB with pH adjusted to 7.3. The fixed brain was then removed and brainstem was dissected with a coronal cut few millimeters nasal to the junction between occipital cortex and cerebellum. The brain block was washed over night in 30% sucrose solution in PBS. Coronal slices (130 µm thick) were cut from the fixed brain block fusing a vibratome microslicer (Linear-Pro7, Dosaka) in ice-cold PBS. The rostral aVCNs were trimmed by hand from the slices. The trimmed sections were then immersed in graded glycerol concentrations of 10−20% at room temperature for 20 min each, followed by 30% at 4°C overnight. The trimmed sections were sandwiched between two metal carriers and then rapidly frozen by a high-pressure freezing machine (HPM010, BAL-TEC, Balzers). Using a freeze etching device (BAF060, BAL-TEC), frozen samples were then fractured into two parts at −115°C, and the fractured faces were replicated by sequential deposition of carbon (thickness: 5 nm from 90° angle), platinum (thickness: 2 nm from 60° angle, unidirectional), and carbon again (thickness: 20 nm from 90° angle). After thawing, the tissue debris attached to the replica was digested with gentle shaking at 80°C for 18 h, in a solution containing 2.5% SDS, 20% sucrose, and 15 mm of Tris-HCl with pH set to 8.3. The replicas were washed 3 × 10 min in wash buffer (0.1% Tween 20, 0.05% BSA, and 0.05% NaN_3_ in TBS, pH 7.4), and then the non-specific binding sites were blocked with 5% BSA in wash buffer for 1 h at 4°C.

For multiple immunolabeling against Ca_V_2.1 and AZ proteins, replicas were first incubated with guinea pig anti-Ca_V_2.1 antibody (8 µg/ml in 1% BSA; Frontier Institute AB_2571851) at 15°C for 3 d, then with anti-guinea pig secondary antibodies conjugated with 10-nm gold particles (1:30 diluted in 5% BSA; British Biocell International) at 15°C overnight, followed by incubation with a cocktail of rabbit anti-AZ proteins antibodies (anti-ELKS at 2 µg/ml in 1% BSA; gift from Dr. Toshihisa Ohtsuka raised against rat ELKS aa117-142, anti-Neurexin at 4 µg/ml in 1% BSA; gift from Dr. Masahiko Watanabe raised against aa1499–1507 and anti-RIM at 4 µg/ml in 1% BSA; Synaptic Systems 140203) at 15°C overnight, and finally with anti-rabbit secondary antibodies conjugated with 5-nm gold particles (1:30 diluted in 5% BSA; British Biocell International) at 15°C overnight. After immunolabeling, replicas were rinsed three times with 0.05% BSA in TBS, washed with TBS and distilled water, and mounted on formvar-coated copper grids.

The labeled replicas were imaged using a Tecnai-12 transmission electron microscope (FEI; AV 120 kV). To obtain a planar view for quantitative measurement of immuno-gold particle number and densities, profile of synaptic structures (AZ and PSD) were tilted in the electron beam. IMP-clusters representing PSDs were manually demarcated and the area was measured using ImageJ software (Rubio et al., 2017). AZ areas were marked by hand with the experimenter being blinded to the identity of the two genotypes. Quantitative analysis of immune-gold particles was done using an in-house software tool, Gold Particle Detection and Quantification (Luján et al., 2018). To define clusters of gold particles the threshold for the distance between particles belonging to the same cluster was calculated as µ + 2σ, where µ and σ are the mean and SD obtained from a Gaussian fit to the distribution of nearest neighbor distances (NNDs) between particles. µ + 2σ was 43.02 and 43.92 nm for Ca_V_2.1 gold particles in RIM-BP2 WT and KO, respectively. We set the threshold at 40 nm to match the value used in a previous analysis (Miki et al., 2017). The distances between particles were measured from their centers of mass, and the minimum number of particles required to form a cluster was set to three. We additionally compared our “real” distribution of NNDs between gold particles and their clustering to 500 random generated by Monte Carlo simulations (as described in Luján et al., 2018; Kleindienst et al., 2020) to confirm that the clusters visualized through our analysis are not generated by chance.

### High-pressure freezing, freeze substitution, and electron tomography

Parasagittal slices from cochlear nuclei were obtained as described for slice electrophysiology. Slices containing the cochlear nucleus were trimmed and mounted onto type A specimen carriers (Leica Microsystems) filled with cutting solution. The flat side of the type B carriers (Leica Microsystems) was dipped in 1-hexadecene (Sigma-Aldrich) and placed onto the type A carriers. Samples were frozen immediately using a HPM100 (Leica Microsystems) and transferred into liquid nitrogen. Freeze-substitution was performed in an EM AFS2 (Leica Microsystems) according to Wong et al. (2014). The slices were incubated in 0.1% (w/v) tannic acid in acetone at −90°C for 4 d and afterward washed three times for 1 h each in acetone at −90°C. 2% (w/v) osmium tetroxide in acetone was applied and incubated for 40.4 h. During that time the temperature was raised slowly to 4°C (10°C/h). At 4°C, osmium tetroxide was removed, and the samples were washed with acetone three times and brought to room temperature. Slices were infiltrated in epoxy resin (Agar-100 kit, Plano; epoxy/acetone 1:1 3–6 h; 100% epoxy overnight). Finally, samples were further incubated in fresh 100% epoxy and placed in embedding molds.

After polymerization for 48 h at 70°C, excess resin was removed with a fine file (DiAtome) and the block was trimmed to a pyramid using a razor blade. To check the region and the structural preservation, 65-nm ultrathin sections were cut with a diamond knife (DiAtome) using an EM UC7 (Leica Microsystems) ultramicrotome. Sections were collected on formvar-coated copper slot grids (Athene, Plano, for ultrathin sections). For electron tomography, 250-nm semi-thin sections were obtained and collected on mesh grids (100 mesh; Athene, Plano, for semi-thin sections). Poststaining was performed with Uranyless (EMS) for 20 min.

The region and quality of the tissue was checked at 80 kV using a JEM1011 transmission electron microscope (JEOL) equipped with a GatanOrius 1200A camera (Gatan). Electron tomography was essentially performed as described previously (Jung et al., 2015; Wong et al., 2014); 10-nm gold beads (British Bio Cell/Plano) were applied to both sides of the stained grids. Big synaptic terminals BCs were identified, and tilt series from endbulb AZs were acquired at 200 kV using a JEM2100 transmission electron microscope (JEOL) mostly from −60° to +60° with a 1° increment at 15,000× using the Serial-EM software package with an image pixel size of 0.95 nm (Mastronarde, 2005). Tomograms were generated using the IMOD package etomo (Kremer et al., 1996).

Only asymmetric synapses with clearly identifiable PSDs were analyzed. However, in high-pressure frozen samples, PSDs appear less electron-dense compared to chemical fixed synapses. Only AZs that showed a PSD and a clear synaptic cleft, originating from large presynaptic terminals were analyzed to exclude inhibitory synapses.

Tomograms were segmented semi-automatically using 3dmod (Kremer et al., 1996). The AZ membrane was manually segmented every 15 virtual sections for five consecutive virtual sections and then interpolated across the *z*-stack, following the extent of the PSD and the parallel synaptic cleft. Moreover, virtual sections were corrected manually after interpolation. The total surface area of this object was then divided by two to calculate the AZ area.

SVs were reconstructed at their maximum projection and the sphere size was adjusted for each vesicle. The smallest distances from the outer leaflet of the SV membrane to the inner leaflet of the AZ membrane were measured and SVs in contact with the AZ membrane were defined as morphologically docked SVs (0- to 2-nm distance). Moreover, all vesicles within 200 nm of the AZ were quantified and categorized in 20-nm bins. The radii of the SVs were determined with the program “imodinfo” of the IMOD software package and the diameters were calculated. For quantification of lateral distances of docked SVs, models of tomogram top views with docked SVs were imported in ImageJ and the center of the captured AZ area was defined by setting two diagonal lines from respective edges of the AZ. The crossing point was defined as the center and the distances were measured from the outer membrane of the SV toward the center point.

### Experimental design and statistical analysis

We investigated the role of RIM-BP2 in synaptic transmission at the endbulb of Held both functionally and morphologically. We used patch-clamp electrophysiology in acute mouse brains slices to assess the Ca^2+^ influx at the presynapse by direct presynaptic terminal recordings. Afferent fiber stimulation was used to assess synaptic strength, synaptic plasticity, SV pool size, and SV release and recovery dynamics. We employed *in vivo* extracellular recordings from single units of BCs in live anesthetised animals to assess the effect of RIM-BP2 deletion on sound processing. To analyze the molecular composition at the endbulb AZs, we employed confocal and STED imaging of immunostained mouse brain slices. We used electron tomography, and immuno-electron microscopy to assess the ultrastructure of the endbulb synapse–SV complement at the AZ, and spatial coupling of SV and Ca^2+^ channels.

Electrophysiology data were analyzed using Igor Pro (Wavemetrics), MATLAB (MathWorks), Mini Analysis (Synaptosoft Inc.), and GraphPad Prism software. Synaptic delay was calculated as the time between the start of stimulus (voltage output of the amplifier as dictated by the experiment protocol) and the time when the respective EPSC response reached 10% of its peak amplitude.

Confocal images were analyzed using ImageJ software, Imaris (Bitplane AG), and MATLAB (MathWorks). STED images of Ca_V_2.1 channels were analyzed using custom Igor pro scripts. Single plane slices of the top (coverslip proximal) and/or bottom (distal from coverslip) membranes of BCs were imaged to capture the Ca_V_2.1 spots on a flat surface top view and enable 2D Gaussian fitting. The Ca_V_2.1 spots that were simultaneously closely juxtaposed to puncta of both Homer1 and Bsn were fitted with Igor Pro's 2D Gaussian function.

Figures were assembled for display using Adobe Illustrator (Adobe Systems). Unless reported otherwise, statistical significance between groups was determined by either unpaired Student's *t* test (in case of normally distributed data with comparable variances between the groups) or Mann–Whitney *U* test (when data distribution did not satisfy the criteria). Normality of distribution was tested with Jarque–Bera test and variances were compared with *F* test. Data are presented as mean ± SEM when normally distributed data with comparable variances between the groups, and otherwise as box and whisker plots showing grand median (of the means of all recordings), lower/upper quartiles, 10–90th percentiles; *p < 0.05, **p < 0.01, ***p < 0.001, and ****p < 0.0001.

### Hierarchical Bayesian modeling

Data of Nearest Neighbor Distances (NNDs) were analyzed with a hierarchical Bayesian model (Kruschke and Vanpaemel, 2015). The γ distribution was chosen as a continuous probability distribution with a low number of parameters and a positive domain. Appropriate to the statistical model, genotypes were used as a fixed effect, while animals were used as a random effect. For this experiment, three animals per genotype were used. The priors for each parameter were set as the default of brm function (RStudio), except for β-coefficients (overall fixed effects for model intercepts) and shapes. Specifically, we used a half-Cauchy prior with location 0 and scale 5 on β-coefficients, and with location 0 and scale 2 on shapes. The modeling was performed using the R language (version 4.0.2) on RStudio (version 1.3.1056) with brms (version 2.13.3) and rstan (version 2.21.1) packages (Buerkner, 2017). Models were estimated via Markov Chain Monte Carlo (MCMC) sampling, running four parallel chains for 10,000 iterations each with a 5000-iteration burn-in. The model comparison was assessed by a Bayes factor and wide-applicable Akaike Information Criteria (WAIC Watanabe, 2010) with models in which the fixed effect had been dropped (null models) from the original models (full models).

## Results

### Deletion of RIM-BP2 impairs synchronous transmitter release at the endbulb of held synapse

**Figure 1.**
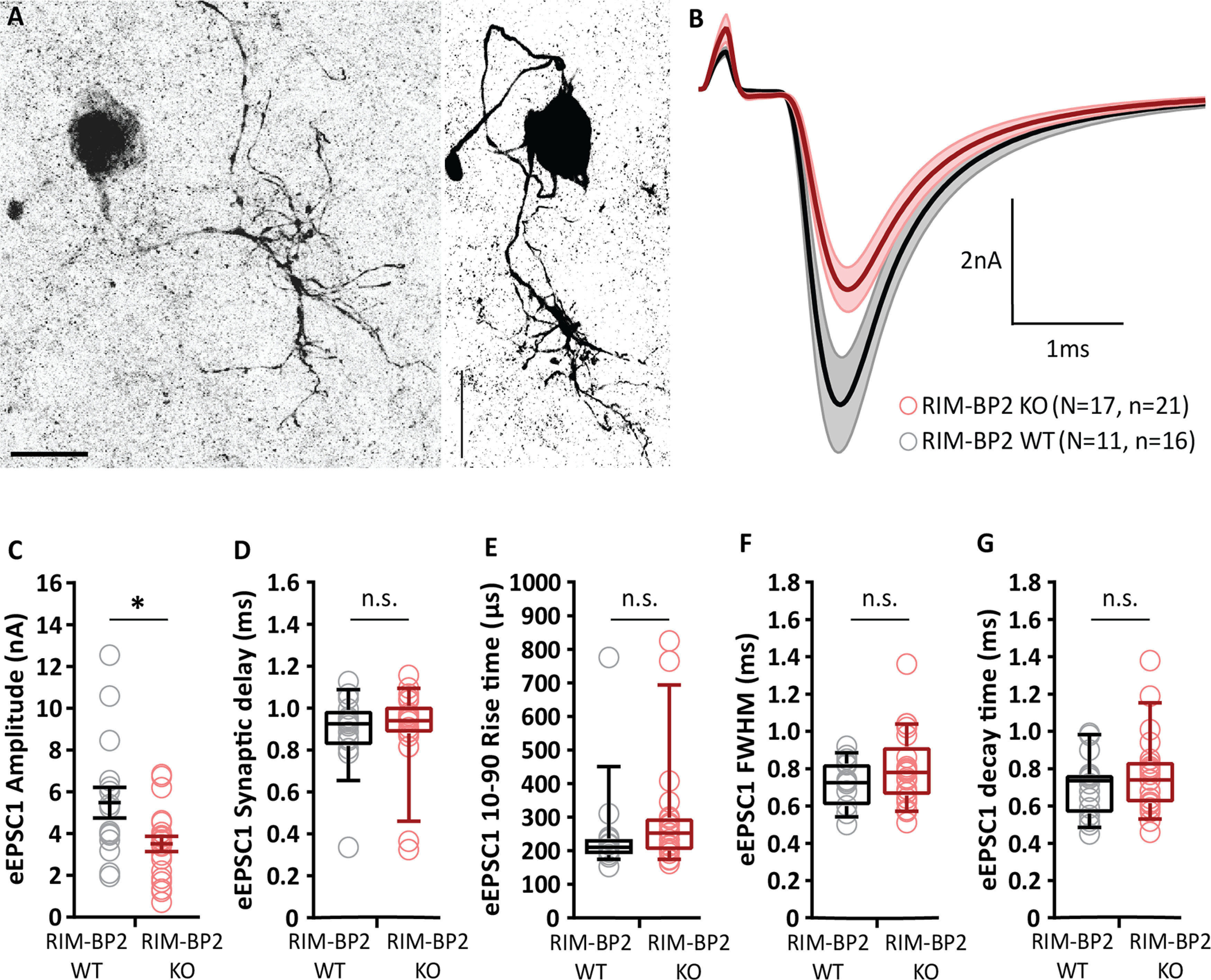
Reduced amplitude of evoked EPSCs in RIM-BP2-deficient endbulb synapses. ***A***, BCs (left and right panels showing dye-filled BCs) were distinguished from stellate cells (another major cell type in the aVCN) by the faster kinetics of their postsynaptic currents (Isaacson and Walmsley, 1995) and their characteristic short-term plasticity (Chanda and Xu-Friedman, 2010). In addition to such functional identification, each recorded cell was filled with fluorescent dye Alexa Fluor 488 via the patch pipette for morphologic distinction. BCs are spherical in appearance with one primary dendrite terminating in a dense bush-like dendritic tree (Wu and Oertel, 1984), distinct from stellate cells, which are asymmetrical in shape and have multiple dendrites branching off in various directions giving them a star-like appearance. Scale bars: 20 µm. ***B***, Average traces (±SEM) of eEPSCs in RIM-BP2 KO (red) and WT (black) endbulbs. ***C***, Reduced eEPSC amplitude in RIM-BP2 KO compared to WT. ***D–G***, No significant differences in eEPSC kinetics: (***D***) synaptic delay, (***E***) 10–90% rise time, (***F***) full width at half-maximum (FWHM), and (***G***) decay time. For an assessment of evoked release in the absence of kynurenic acid and CTZ, see Figure 2. To test whether the observed differences could be accounted for by changes in spontaneous release, sEPSC amplitude and kinetics were compared between genotypes in Figure 3 and Table 1. Data points represent the mean estimate of each BC included in the analysis. Normality was tested with the Jarque–Bera test. Normally distributed data (***C***) are shown as grand mean (of the means of all BCs) ± SEM. Non-normally distributed data (***D*–*G***) are presented as box and whisker plots (grand median of all BC means, lower/upper quartiles, 10–90th percentiles); *p < 0.05, n.s. *p* ≥ 0.05, Mann–Whitney *U* test. *N*: number of animals, *n*: number of BCs.

To determine the functional role of RIM-BP2, we studied synaptic transmission at the endbulb of Held synapse in acute parasagittal slices of the brainstem of constitutive RIM-BP2 KO mice (RIM-BP2 KO; Grauel et al., 2016) recording spontaneous EPSCs (sEPSCs) (no stimulation, no TTX applied) and evoked EPSCs (eEPSCs) from BCs of the aVCN (Fig. 1*A*) at postnatal days 15–21. eEPSCs were elicited by minimal electrical stimulation of the presynaptic ANF by a monopolar electrode placed in the proximity (approximately three cell diameters away) of the recorded BC, whereby each stimulus is aimed to elicit one action potential in one endbulb (Yang and Xu-Friedman, 2008).

**Figure 2.**
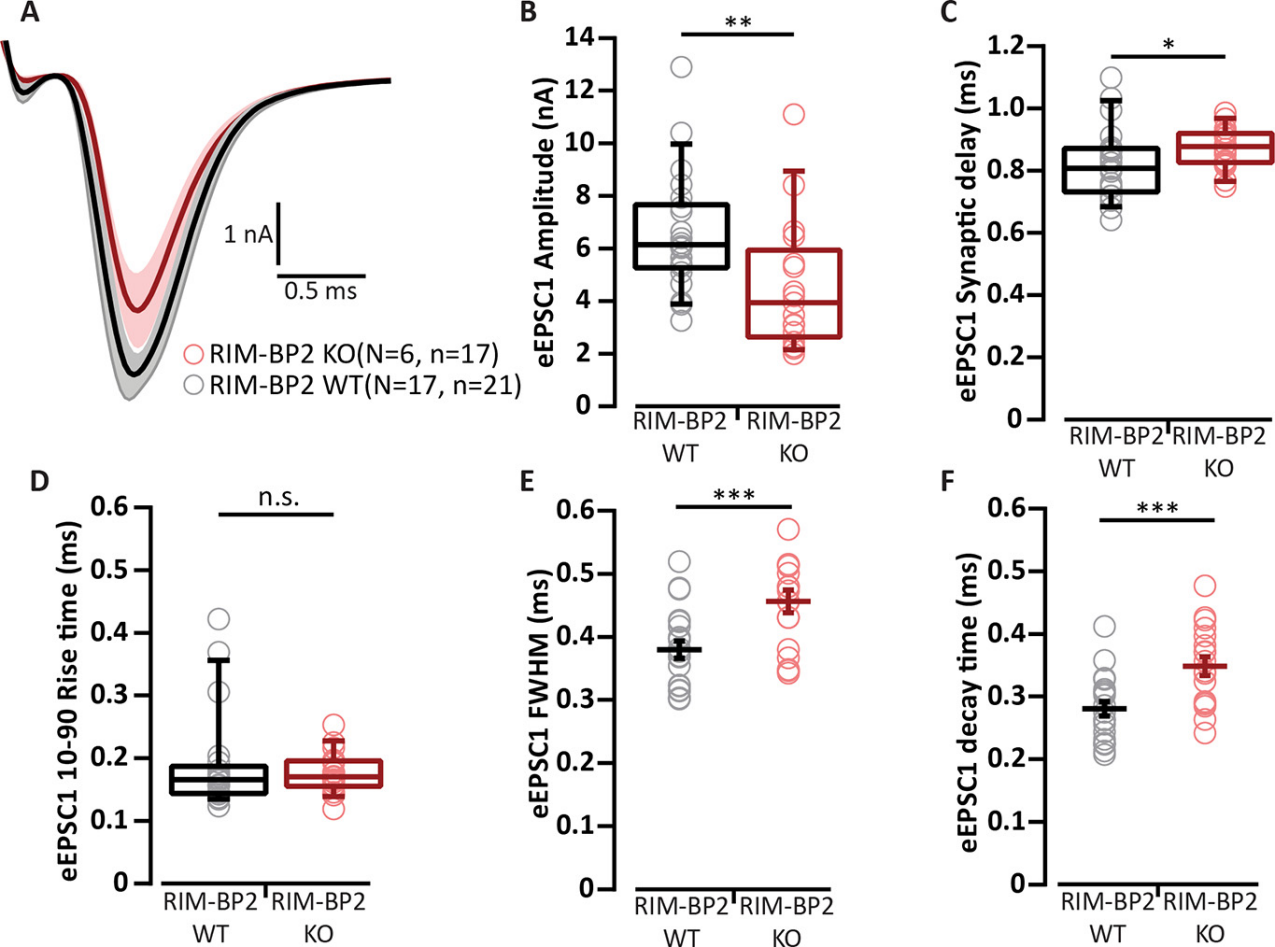
Evoked EPSC recordings in the absence of kynurenic acid and CTZ. ***A***, Average traces (±SEM) of eEPSCs in RIM-BP2 KO (red) endbulbs and WT (black) in the absence of kynurenic acid and CTZ. ***B***, Reduced eEPSC amplitude in RIM-BP2 KO compared to WT. (***C–F***) eEPSC kinetics: (***C***) a larger synaptic delay, (***D***) unchanged 10–90% rise time, (***E***) wider full-width at half-maximum (FWHM), and (***F***) slower decay time. Data points represent the mean estimate of each BC included in the analysis. Normality was tested with the Jarque–Bera test. Normally distributed data (***E***, ***F***) are shown as grand mean (of the means of all BCs) ± SEM, and significance was tested with Student's *t* test. Non-normally distributed data (***B*–*D***) are presented as box and whisker plots (grand median of all BC means, lower/upper quartiles, 10–90th percentiles), and significance was tested with Mann–Whitney *U* test; n.s. *p* ≥ 0.05; *p < 0.05, **p < 0.01, ***p < 0.001. *N*: number of animals, *n*: number of BCs.

Experiments of Figure 1 were performed in the presence of 1 mm kynurenic acid and 100 µm CTZ to avoid saturation and desensitization of AMPA receptors (Chanda and Xu-Friedman, 2010), respectively. The concentration of CTZ used here, was adopted from previous reports at the calyx of Held (Sakaba and Neher, 2001; Thanawala and Regehr, 2013). The former study (Sakaba and Neher, 2001) examined the NMDA current in the presence of 100 µm CTZ and found no evidence for a presynaptic CTZ effect at the calyx of Held. The eEPSC amplitude was reduced in RIM-BP2 KO BCs compared to littermate WT [3.50 ± 0.37 nA (KO) vs 5.48 ± 0.73 nA (WT), *p* = 0.022, Mann–Whitney *U* test; Fig. 1*B*,*C*]. Both the eEPSC synaptic delay [0.89 ± 0.04 ms (KO) vs 0.89 ± 0.2 ms (WT) *p* = 0.5691, Mann–Whitney *U* test; Fig. 1*D*] and 10–90 rise time [297.76 ± 38.47 µs (KO) vs 247.77 ± 61.93 µs (WT) *p* = 0.1650, Mann–Whitney *U* test; Fig. 1*E*] were comparable between the two genotypes. The eEPSC full width at half maximum [0.80 ± 0.04 ms (KO) vs 0.72 ± 0.18 ms (WT) *p* = 0.3119, Mann–Whitney *U* test; Fig. 1*F*] and the decay time [0.77 ± 0.04 ms (KO) vs 0.71 ± 0.18 ms (WT) *p* = 0.4163, Mann–Whitney *U* test; Fig. 1*G*] also remained unchanged. EPSC recordings in the absence of kynurenic acid and CTZ confirmed the eEPSC amplitude reduction [4.54 ± 0.75 nA (KO) vs 6.57 ± 0.47 nA (WT), *p* = 0.004, Mann–Whitney *U* test; Fig. 2*A*,*B*], eliminating the possibility of CTZ obscuring the changes in AMPA receptor composition or inducing membrane potential changes. The eEPSC synaptic delay was longer [0.89 ± 0.02 ms (KO) vs 0.82 ± 0.03 ms (WT) *p* = 0.013, Mann–Whitney *U* test; Fig. 2*C*], 10–90 rise time remained unchanged [0.18 ± 0.01 ms (KO) vs 0.19 ± 0.02 ms (WT) *p* = 0.49, Mann–Whitney *U* test; Fig. 2*D*], and decay kinetics were significantly slower for RIM-BP2 KO BCs under these conditions [full width at half maximum was 0.46 ± 0.02 ms (KO) vs 0.38 ± 0.01 ms (WT) *p* = 0.0005, Student's *t* test, and decay time was 0.35 ± 0.02 ms (KO) vs 0.28 ± 0.02 ms (WT) *p* = 0.0004, Student's *t* test; Fig. 2*E*,*F*].

**Figure 3.**
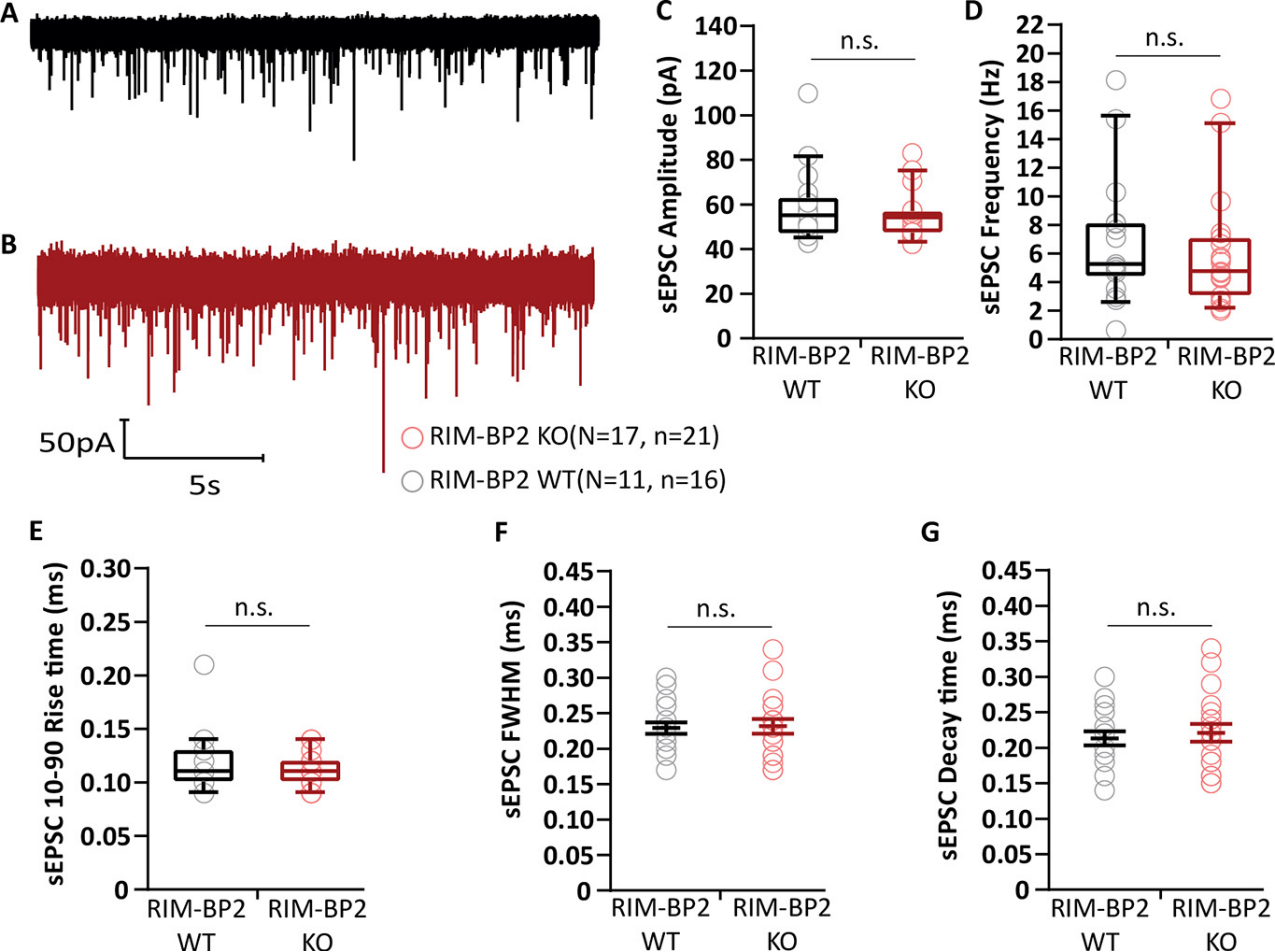
Unaltered amplitude, kinetics and frequency of sEPSCs in RIM-BP2-deficient endbulb synapses. ***A***, ***B***, Representative sEPSCs at WT (***A***, black) and RIM-BP2 KO (***B***, red) endbulbs of held: continuous traces are shown. No significant differences in (**C**) amplitude, (***D***) frequency, (***E***) 10–90% rise time, (***F***) full-width at half-maximum (FWHM), and (***G***) decay time in RIM-BP2-deficient endbulbs. sEPSC were recorded while intracellularly blocking BC action potential generation with QX-314. Each data point represents the mean estimate of each BC included in the analysis. Normality was tested with the Jarque–Bera test. Normally distributed data are depicted as mean (grand average of all BC means) ± SEM (***F***, ***G***; n.s. *p* ≥ 0.05, unpaired Student's *t* test). Non-normally distributed data are presented as box and whisker plots (grand median of all BC means, lower/upper quartiles, 10–90th percentiles; ***C*–*E***); n.s. *p* ≥ 0.05, Mann–Whitney *U* test, *N*: number of animals, *n*: number of BCs.

In order to test for potential changes in the quantal release properties we recorded sEPSCs from BCs (Fig. 3). We did not observe differences in the sEPSC amplitude [54.91 ± 1.46 pA (KO) vs 58.17 ± 3.70 pA (WT), *p* = 0.5781, Mann–Whitney *U* test; Fig. 3*A–C*]. Similarly, the sEPSC frequency [6.02 ± 2.10 pA (KO) vs 9.02 ± 2.40 pA (WT), *p* = 0.3127, Mann–Whitney *U* test; Fig. 3*D*], 10–90 rise time [0.11 ± 0.002 ms (KO) vs 0.11 ± 0.006 ms (WT), *p* = 0.9353, Mann–Whitney *U* test; Fig. 3*E*], full-width at half-maximum [0.23 ± 0.006 ms (KO) vs 0.23 ± 0.008 ms (WT), *p* = 0.9364, Mann–Whitney *U* test; Fig. 3*F*], and decay time [0.22 ± 0.007 ms (KO) vs 0.21 ± 0.010 ms (WT), *p* = 0.8336, Mann–Whitney *U* test; Fig. 3*G*] were unaltered in the RIM-BP2 KO. This was also the case when recording in the absence of kynurenic acid and CTZ (Table 1). Unaltered sEPSCs suggest that the properties of single SV release and of postsynaptic glutamate response are intact in RIM-BP2-deficient endbulb synapses at BCs.

**Table 1.** Unaltered sEPSC at the RIM-BP2-deficient endbulb of Held in the absence of kynurenic acid and CTZ

Parameter	RIM-BP2 WT	RIM-BP2 KO	*p* value
Amplitude (pA)	130.54 ± 12.06	112.48 ± 4.03	0.22, M
10–90% rise time (ms)	0.09 ± 0.003	0.09 ± 0.002	0.85, T
FWHM (ms)	0.18 ± 0.008	0.19 ± 0.004	0.46, T
Decay time (ms)	0.16 ± 0.010	0.17 ± 0.005	0.36, T
Frequency (Hz)	7.65 ± 1.28	8.01 ± 1.09	0.83, T

Data presented as mean (grand average of the means of all BCs) ± SEM. Statistical significance between groups was determined by either unpaired Student's *t* test (in case of normally distributed data with comparable variances between the groups, indicated as T) or Mann–Whitney *U* test (when data distribution did not satisfy the criteria, indicated at M). Normality of distribution was tested with Jarque–Bera test and variances were compared with *F* test. WT *N* = 4; *n* = 10, RIM-BP2 KO *N* = 7; *n* = 19 (*N*: number of animals; *n*: number of BCs).

**Figure 4.**
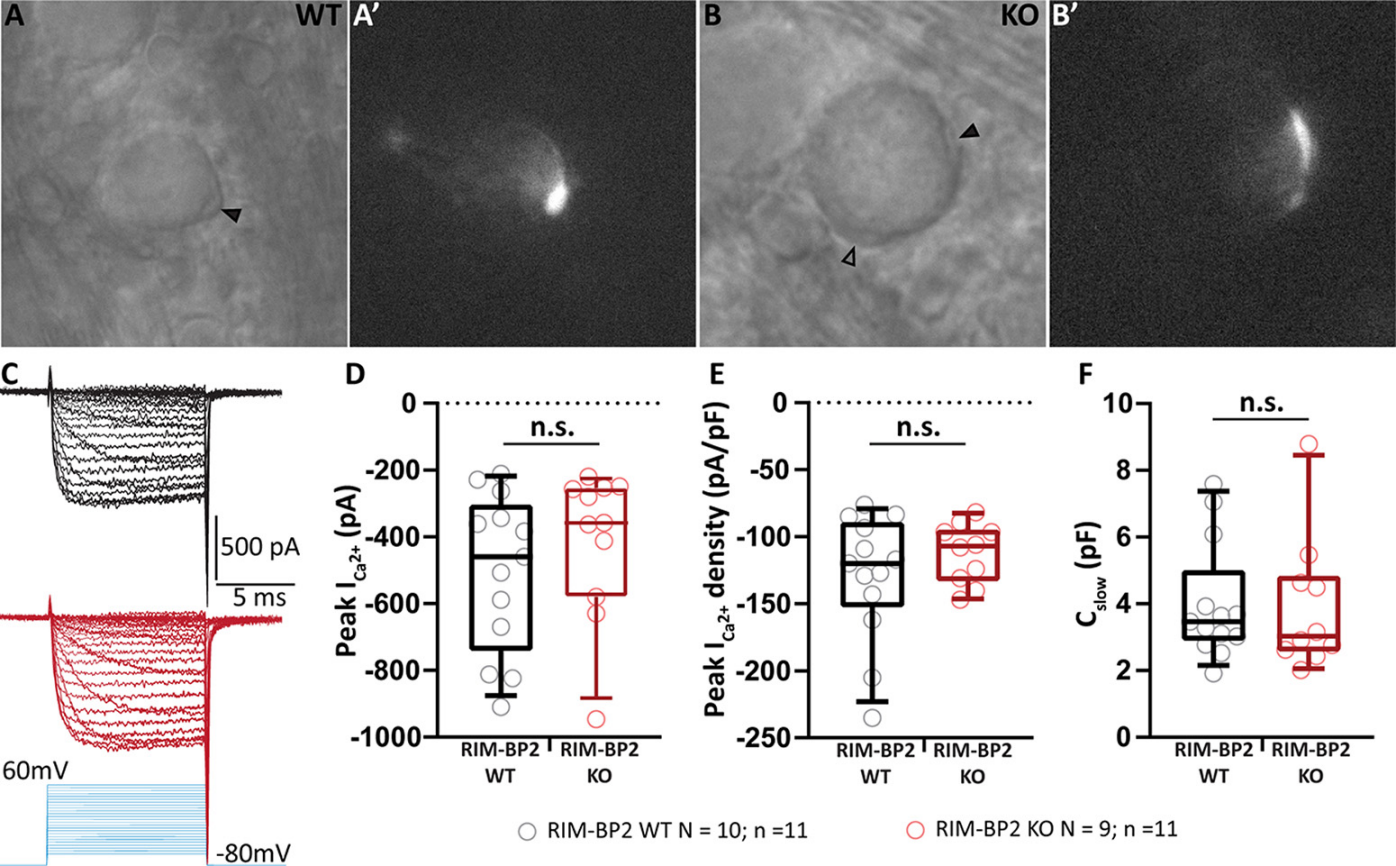
Ca^2+^ influx is unaltered in RIM-BP2-deficient endbulbs. ***A***, ***B***, Bright-field images of the WT (***A***) and RIM-BP2 KO (***B***) BCs under DIC. The recorded endbulb is demarcated by a solid black arrowhead (***A***, ***B***), while another endbulb onto to the same BC is shown by an open black arrowhead (***B***). Each recorded endbulb was filled with Alexa Fluor 488 to confirm that only the presynaptic terminal was accessed, and not the postsynaptic cell. Fluorescent endbulbs are shown in ***A'***, ***B'***. ***C***, Representative traces of presynaptic Ca^2+^ currents in WT (black) and RIM-BP2 KO (red) in response to stepped increase in holding potential (stimulus protocol in blue). ***D–F***, No significant difference was observed between the two genotypes in the peak Ca^2+^ current (***D***), peak Ca^2+^ density (***E***; peak current/C_slow_), and the size of the endbulb (***F***; estimated by C_slow_). Each data point represents the mean estimate of each endbulb included in the analysis. Normality was tested with the Shapiro–Wilk test, and variance with *F* test. All data are presented as box and whisker plots (grand median of all BC means, lower/upper quartiles, 10–90th percentiles). Significance of normally distributed data with equal variances (***D–E***) was tested with Student's *t* test, while non-normally distributed data (***F***) was tested with Mann–Whitney *U* test, n.s. *p* ≥ 0.05, *N*: number of animals, *n*: number of endbulbs of Held.

Quantal size being unaltered, the reduced eEPSC alteration could result from either: (1) an impaired stimulus-secretion coupling because of reduced Ca^2+^ influx and/or altered topography of Ca^2+^ channels (Krinner et al., 2017) and fusion competent SVs (Acuna et al., 2015; Grauel et al., 2016; Luo et al., 2017), or (2) because of an impaired SV priming that has recently been shown to involve RIM-BP2 interaction with Munc13-1 (Brockmann, 2020). We first checked for changes in presynaptic Ca^2+^ influx in the absence of RIM-BP2 using ruptured-patch recordings from the endbulb of Held (Lin et al., 2011) in mice after the hearing onset (postnatal days 13–16; Fig. 4*A*,*B*). We did not observe significant changes in the peak Ca^2+^ current amplitude [413.50 ± 67.17 pA (KO) vs 504.90 ± 65.97 pA, *p* = 0.34, Student's *t* test; Fig. 4*C*,*D*] or peak Ca^2+^ current density in KO endbulbs of Held [112.00 ± 7.06 pA/pF (KO) vs 129.60 ± 13.16 pA/pF, *p* = 0.29, Student's *t* test; Fig. 4*E*]. The size of the endbulbs estimated by the C_slow_ was comparable between the genotypes [3.92 ± 0.64 pF (KO) vs 4.00 ± 0.49 pA, *p* = 0.65, Mann–Whitney *U* test; Fig. 4*F*].

**Figure 5.**
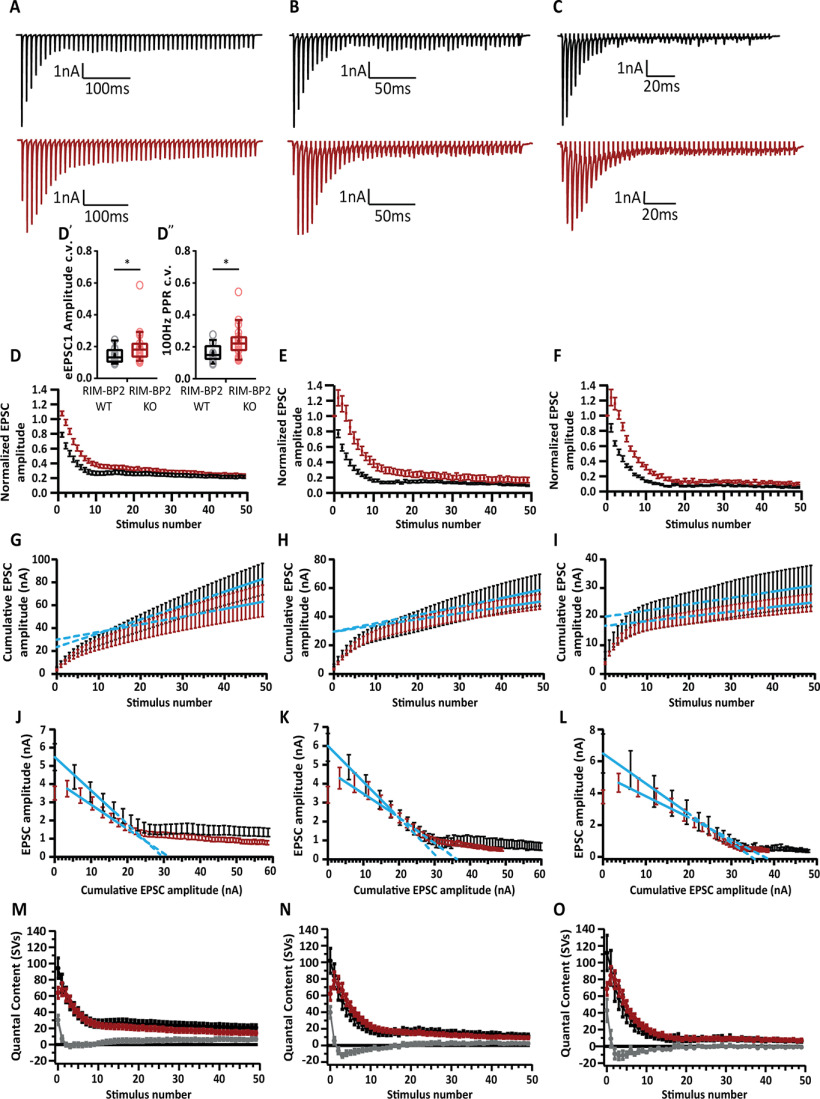
Analysis of release probability (P_r_) as well as the size and dynamics of the RRP. ***A–C***, Representative traces of eEPSCs in response to trains of 50 action potentials delivered at frequencies of 100 Hz (***A***), 200 Hz (***B***), and 333 Hz (***C***) recorded from WT (top, black traces) and RIM-BP2 KO (bottom, red traces). Note the characteristic fast short-term depression of WT BC EPSCs that is altered in the mutant. The mutant BCs show a delayed short-term depression with the first EPSC amplitude not being the largest in the train, indicating that the naive mutant synapse releases most of its vesicles later in the train. ***D*–*F***, Average EPSC amplitudes, normalized to the first EPSC of the train plotted against the stimulus number. ***D'***, For each cell included in the 100-Hz dataset, the trial-to-trial variability of SV release was assessed by calculating the eEPSC1 amplitude c.v. The KO shows significantly higher variability than the WT. ***D''***, The trial-to-trial variability of P_r_ was similarly assessed using the PPR c.v. as a proxy. Here, again, the KO endbulbs display higher PPR variability than WT endbulbs. ***G–I***, To estimate the size of the RRP, the rate of vesicle replenishment and the P_r_ using the SMN method, the EPSC amplitudes of the 100-Hz (***G***), 200-Hz (***H***), and 333-Hz (***I***) trains were plotted cumulatively against the stimulus number. The linear fit (solid blue line) to the last ten steady-state values was back-extrapolated to the *y*-axis (dotted blue line). The *y*-intercept value, divided by the average sEPSC amplitude yields the number of vesicles in the RRP. To calculate P_r_, the vesicle content of eEPSC1 is divided by the size of the RRP. The slope of the linear fit approximates the rate of vesicle replenishment during the train. Quantitative analysis is further elaborated in Table 2. ***J–L***, To estimate the RRP size and P_r_ using the EQ method, absolute EPSC amplitudes were plotted against the cumulative amplitude of all the EPSCs preceding the corresponding EPSC. The linear fit to the first three to five points for the 100-Hz (***J***), 200-Hz (***K***), and 333-Hz (***L***) trains (solid blue line) was forward extrapolated (dotted blue line) to the *x*-axis. Dividing the *x* intercept value by the average sEPSC size yields the size of the RRP, while the slope of the linear fit defines the P_r_. To assess whether the reduced P_r_ results from fewer superprimed SVs in the RRP, a subtraction analysis was performed. We first divided the average eEPSC amplitudes by the average sEPSC amplitude to calculate the SV number released during each eEPSC (quantal content) of the averaged responses to 100-, 200-, and 333-Hz train stimulation. Traces plot the quantal content released during each one of 50 eEPSCs in 100-Hz (***M***), 200-Hz (***N***), and 333-Hz (***O***) trains against the stimulus number. After subtracting the quantal content for each RIM-BP2 KO eEPSC from the respective WT eEPSC, we plot the subtraction curves (gray curves at the bottom of each panel). For an assessment of P_r_ and RRP dynamics in the absence of kynurenic acid and CTZ along with the changes of RIM-BP2 KO short-term plasticity in response to increased [Ca^2+^]_e_, see Figure 6. For 100 Hz: RIM-BP2 WT *N* = 14; *n* = 16, RIM-BP2 KO *N* = 17; *n* = 21. For 200 Hz: RIM-BP2 WT *N* = 13; *n* = 16, RIM-BP2 KO *N* = 13; *n* = 16. For 333 Hz: RIM-BP2 WT *N* = 6; *n* = 12, RIM-BP2 KO *N* = 14; *n* = 17. *N*: number of animals; *n*: number of BCs.

**Figure 6.**
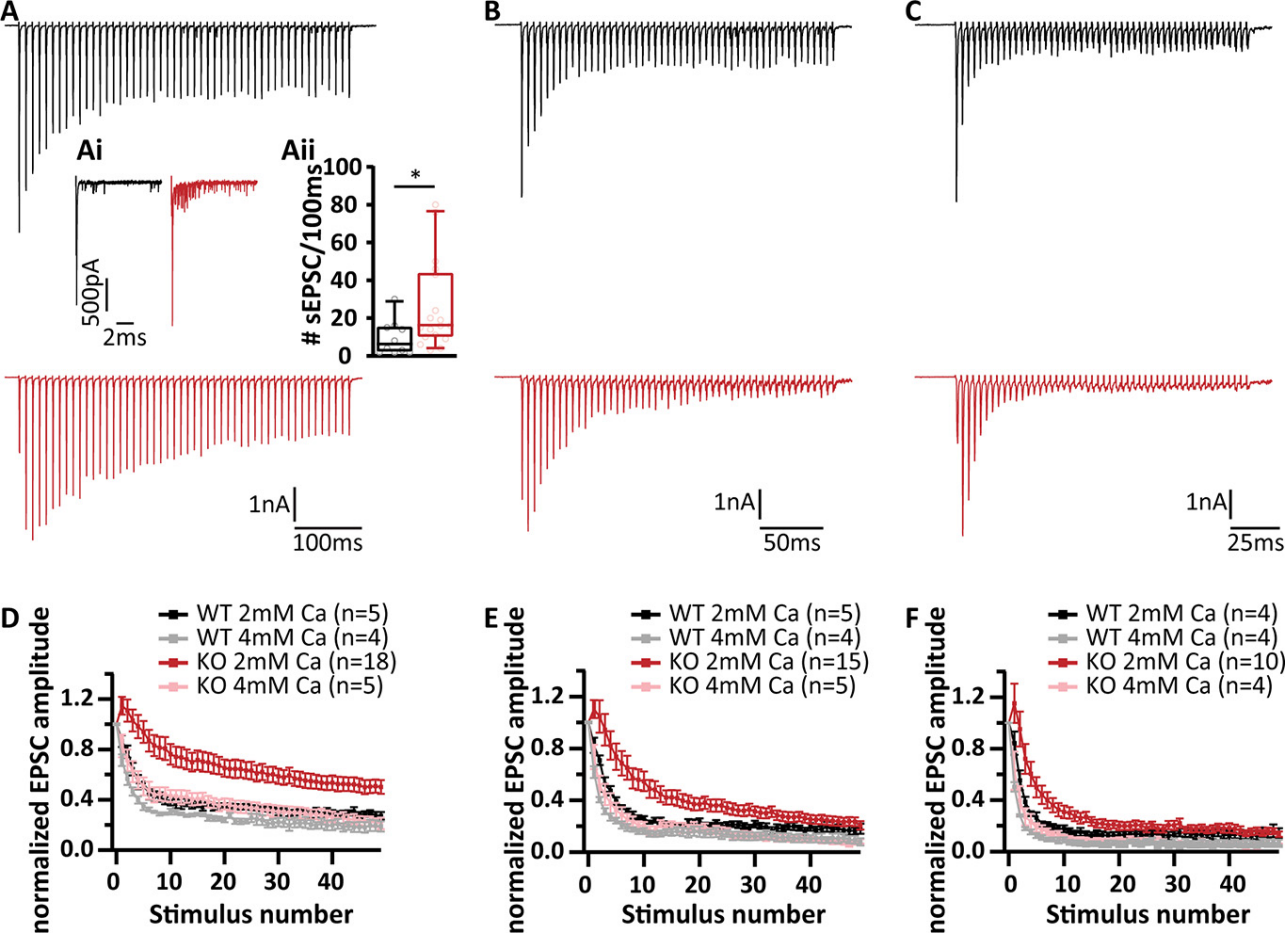
Short-term depression replaced by facilitation at endbulbs of Held in RIM-BP2 KO mice in the absence of kynurenic acid and CTZ. Representative traces of eEPSCs in response to trains of 50 action potentials delivered at frequencies of 100 Hz (***A***), 200 Hz (***B***), and 333 Hz (***C***) recorded from WT (top, black traces) and RIM-BP2 KO (bottom, red traces). Inset (***Ai***, ***Aii***), Asynchronous release calculated as the number of sEPSC events within 100 ms following the synchronous release elicited by a train of 50 pulses delivered at 100 Hz. A significantly higher asynchronous release was observed in the RIM-BP2 KO (red, 25.67 ± 5.55 sEPSC/100 ms) as compared to the WT (black, 9.60 ± 2.90 sEPSC/100 ms; *p* = 0.02, Mann–Whitney *U* test). Note the characteristic fast short-term depression of WT BC EPSCs that is altered in the mutant. The mutant BCs show a delayed short-term depression with the first EPSC amplitude not being the largest in the train, indicating that the naive mutant synapse releases most of its vesicles later in the train. This effect is more pronounced in the higher frequencies of stimulation, as is demonstrated when the EPSC amplitudes, normalized to the first EPSC of the train are plotted against the stimulus number (***D*–*F***). This effect was abolished when extracellular Ca^2+^ was increased to 4 mm (WT traces in gray and KO traces in pink).

Next, we investigated whether eEPSC alteration is caused by a reduction in release probability or altered vesicle pool dynamics. We used high-frequency stimulation to assess short-term plasticity, release probability as well as the size and dynamics of the readily releasable pool (RRP) during quasi physiological regimes of synaptic transmission. Fifty consecutive stimuli were delivered at 100, 200, and 333 Hz [in the presence of 1 mm kynurenic acid and 100 µm CTZ (Fig. 5); in the absence of both drugs (Fig. 6)]. Different from the prominent short-term depression typical for the endbulb of Held (Oleskevich and Walmsley, 2002; Yang and Xu-Friedman, 2008), RIM-BP2-deficient endbulb synapses showed an initial facilitation followed by slower depression (Figs. 5, 6; Table 2). Facilitation was evident from the paired pulse ratio (PPR) >1 in RIM-BP2-deficient endbulb synapses for all interstimulus intervals tested, while PPR was consistently ∼0.8 in WT (100 Hz Mann–Whitney *U* test, 200 Hz: Mann–Whitney *U* test, 333 Hz: Student's *t* test, *p* < 0.05; Table 2). eEPSC1 and PPR were not only altered on average, but also showed greater variance. Consistent with a prior analysis of RIM-BP1/2-deficient calyx of Held (Acuna et al., 2015), the trial-to-trial coefficient of variation (c.v.) of eEPSC1 was significantly increased compared to WT [0.18 ± 0.02 (KO) vs 0.14 ± 0.01 (WT), Mann–Whitney *U* test *p* = 0.035; Fig. 5*D'*]. We also assessed the trial-to-trial variability of PPR as a proxy for P_r_ variability and found the c.v. also increased in the RIM-BP2-edicient endbulb synapses exposed to 100-Hz trains of stimulation [0.22 ± 0.02 (KO) vs 0.17 ± 0.02 (WT), Mann–Whitney *U* test *p* = 0.015; Fig. 5*D''*]. Interestingly, at 200 and 333 Hz stimulation, there was no significant difference in PPR c.v. (Mann–Whitney *U* test *p* = 0.8039; data not shown). In contrast to (Acuna et al., 2015), we did not find a significantly more variable EPSC rise time in the absence of RIM-BP2. The extent of depression assessed as EPSC_30–50_/EPSC_max_ (Figs. 5, 6; Table 2) tended to be less in RIM-BP2-deficient endbulb synapses without reaching statistical significance. Release probability (P_r_) as well as the size and dynamics of the RRP were estimated by applying two variants of the cumulative analysis to the EPSC trains (Fig. 5): Schneggenburger–Meyer–Neher (SMN) method (Schneggenburger et al., 1999) and Elmqvist and Quastel (EQ) method (Elmqvist and Quastel, 1965; for review, see Neher, 2015). Both methods revealed a significant reduction of P_r_ in RIM-BP2 KO synapses while RRP size and the replenishment rate were not significantly altered (Table 2). In an attempt to address the question whether the reduced release probability reflects a smaller complement of high release probability SVs (“tightly docked” or “superprimed” SVs; Neher and Brose, 2018), we followed a previously described analysis that found a reduction of superprimed SVs on genetic deletion of all rab3 isoforms (Schlüter et al., 2006). We subtracted the quantal content of each RIM-BP2 KO response from the respective RIM-BP2 WT response during train stimulation (Fig. 5*M–O*). This analysis revealed the strongest difference between the two genotypes occurs at the beginning of the train, where the RIM-BP2 KO endbulbs release 30–40 SVs less than the RIM-BP2 WT, which is consistent with a reduction of high release probability (superprimed) SVs in the absence of RIM-BP2. The difference vanishes already after the first two eEPSCs, when the subtraction curves for 200 and 333 Hz cross the zero line, indicating that release from KO-synapses is actually slightly larger than that from WT. Possible reasons for this small difference include (1) protracted release of low release probability SVs that could result from impaired Ca_V_-release site coupling and (2) residual desensitization that is more prominent for RIM-BP2 WT synapses with larger eEPSCs (despite 100 µm CTZ and 1 mm kynurenic acid). During the steady state response to the train stimulation, the WT synapses tended to release more SVs than the KO (average difference: six SVs for 100 Hz, three SVs for 200 Hz, no difference for 333 Hz). After cessation of the stimulus train, RIM-BP2 KO synapses showed enhanced asychronous release (Fig. 6*Ai* and *Aii*).

**Table 2.** Analysis of P_r_ as well as the size and dynamics of the RRP

Stimulation frequency	Parameter	RIM-BP2 WT	RIM-BP2 KO	*p* value
100 Hz	eEPSC1 amplitude (nA)	5.48 ± 0.73 (4.7)	3.5 ± 0.37 (3.6)	**0.022, M**
	tau (ms)	33.60 ± 3.64 (27.2)	56.95 ± 3.76 (58.03)	**0.0001, T**
	eEPSC_30–50_ (nA)	1.29 ± 0.22 (1.09)	0.87 ± 0.12 (0.84)	0.1015, M
	eEPSC_30–50_/EPSC_max_	0.23 ± 0.02 (0.21)	0.24 ± 0.03 (0.24)	0.8205, T
	Replenishment	2.09 ± 0.35 (1.79)	1.44 ± 0.20 (1.2)	0.1015, T
	RRP_SMN_ (SVs)	398.60 ± 72.50 (341.56)	455.61 ± 63.00 (446.32)	0.4038, M
	RRP_EQ_ (SVs)	575.11 ± 102.11 (504.25)	593.62 ± 73.45 (610.12)	0.6826, M
	P_r SMN_	0.26 ± 0.02 (0.28)	0.17 ± 0.01 (0.15)	**0.0004, T**
	P_r EQ_	0.2 ± 0.02 (0.21)	0.14 ± 0.01 (0.13)	**0.0336, T**
	PPR	0.79 ± 0.03 (0.72)	1.08 ± 0.10 (0.97)	**0.0004, M**
200 Hz	tau (ms)	33.05 ± 3.04 (32.46)	70.51 ± 5.85 (65.43)	**<0.0001, M**
	eEPSC_30–50_ (nA)	0.76 ± 0.13 (0.70)	0.59 ± 0.07 (0.56)	0.0608, M
	eEPSC_30–50_/EPSC_max_	0.13 ± 0.01 (0.14)	0.17 ± 0.03 (0.13)	0.0608, M
	Replenishment	2.16 ± 0.37 (1.98)	1.53 ± 0.24 (1.45)	0.1756, T
	RRP_SMN_ (SVs)	479.11 ± 110.23 (351.27)	544.42 ± 77.85 (545.76)	0.3168, M
	RRP_EQ_ (SVs)	554.34 ± 123.03 (441.21)	771.93 ± 108 (663.17)	0.0883, M
	P_r SMN_	0.25 ± 0.02 (0.26)	0.14 ± 0.01 (0.13)	**<0.0001, M**
	P_r EQ_	0.22 ± 0.02 (0.20)	0.13 ± 0.01 (0.13)	**0.0002, T**
	PPR	0.81 ± 0.05 (0.79)	1.28 ± 0.10 (1.21)	**<0.0001, M**
333 Hz	tau (ms)	19.34 ± 2.50 (19.77)	39.97 ± 3.92 (40.97)	**0.0002, T**
	eEPSC_30–50_ (nA)	0.45 ± 0.09 (0.38)	0.48 ± 0.08 (0.41)	0.1523, M
	eEPSC_30–50_/EPSC_max_	0.07 ± 0.01 (0.07)	0.10 ± 0.02 (0.08)	0.6106, M
	Replenishment	1.24 ± 0.21 (1.22)	1 ± 0.25 (0.89)	0.1656, M
	RRP_SMN_ (SVs)	345.21 ± 96.42 (253.09)	306.16 ± 36.91 (331.58)	0.6471, M
	RRP_EQ_(SVs)	595.52 ± 155.74 (450.19)	674.17 ± 76.94 (787.59)	**0.0043, T**
	P_r EQ_	0.23 ± 0.03 (0.21)	0.15 ± 0.01 (0.15)	**0.0022, T**
	PPR	0.8 ± 0.05 (0.84)	1.28 ± 0.11 (1.21)	**0.0009, T**

Tau (τ): time constant of single exponential fit to the decay of eEPSC during the stimulation train. eEPSC_30–50_/EPSC_max_: average amplitude of the last 20 EPSCs (30–50) in the train, normalized to the amplitude of the largest EPSC of the train. Replenishment: rate of vesicle replenishment. RRP: readily releasable pool. P_r_: release probability. PPR: paired pulse ratio, amplitude of the second EPSC of the train normalized to the amplitude of the first EPSC. Data are presented as mean ± SEM, and medians are shown in parentheses. Normality of data distribution was tested with Jarque–Bera test, and the variances were compared with *F* test. Statistical significance of differences was assessed with unpaired Student's *t* test (shown in the table as T), when the data satisfied the criteria of normality and variance comparability. When the data did not satisfy these criteria, Mann–Whitney *U* test was used instead (shown in the table as M); *p* < 0.05, set as threshold for statistical significance. Statistically significant differences are shown in bold.

**Figure 7.**
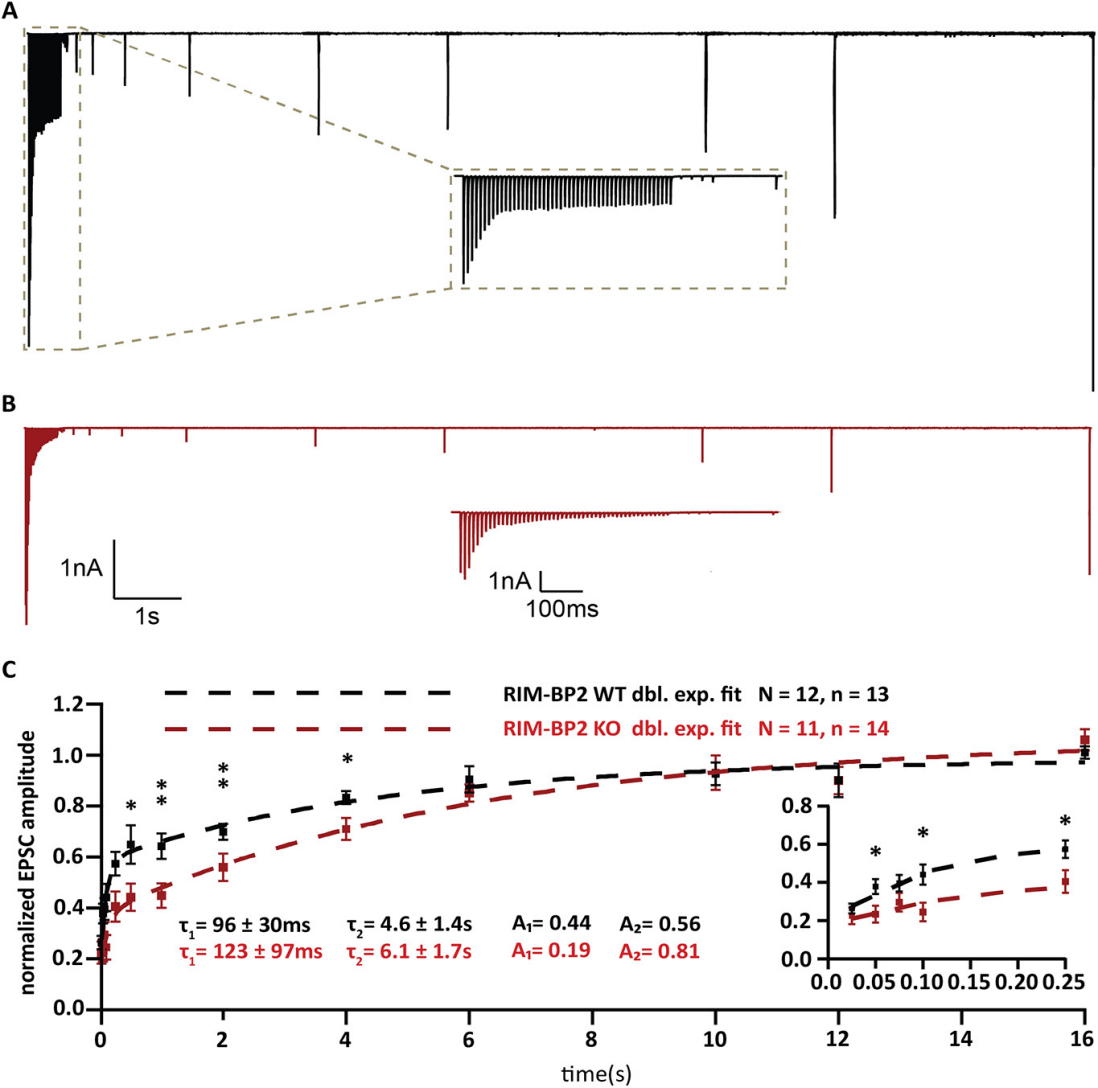
Slowed RRP recovery in RIM-BP2-deficient endbulb synapses. ***A***, ***B***, Representative traces of RIM-BP2 WT (***A***) and RIM-BP2 KO (***B***) illustrate the recovery experiment. After a 100-Hz conditioning train of 50 stimuli, single test pulses were delivered at time intervals of (in ms) 25, 50, 75, 100, 250, 500 (further intervals in s), 1, 2, 4, 6, 10, 12, and 16. To assess recovery, the EPSC amplitude in response to the test pulse is normalized to the first EPSC amplitude of the conditioning train. Insets (***A***, ***B***) show the time course of recovery during the first five test stimuli in subsecond detail. ***C***, Recovery is plotted as mean ± SEM EPSC amplitude in response to test pulses normalized to the first EPSC amplitude of the conditioning train. The double exponential fits are represented by the dashed lines for RIM-BP2 WT (black) and RIM-BP2 KO (red). The time constants (τ) and fractional contributions (***A***) of fast (τ_1_, A_1_) and slow (τ_2_, A_2_) recovery components are provided on the graph. Inset shows the first five responses in detail. Normality was tested with Jarque–Bera test. Statistical significance between groups was tested with Mann–Whitney *U* test; **p < 0.01, *p < 0.05. *N*: number of animals, *n*: number of BCs.

In order to further scrutinize RRP dynamics, we studied the recovery from short-term depression, by measuring eEPSC amplitudes elicited by single stimuli presented at varying time intervals after a conditioning 100-Hz train of 50 pulses (Fig. 7). Recovery is displayed as the eEPSC amplitudes normalized to the amplitude of the first eEPSC of the conditioning train (Fig. 7*A*,*B*): RIM-BP2-deficient endbulb synapses showed a major reduction of fast recovery. The time course was fitted with a double exponential function revealing the following tau values and fractional contributions of the fast and slow components of recovery: τ_1_ = 123 ± 97 ms, A_1_ = 0.19; τ_2_ = 6.1 ± 1.7 s, A_2_ = 0.81 for RIMBP2 KO and τ_1_ = 96 ± 30 ms, A_1_ = 0.44; τ_2_ = 4.6 ± 1.4 s, A_2_ = 0.56 for WT. (Fig. 7*C*).

**Figure 8.**
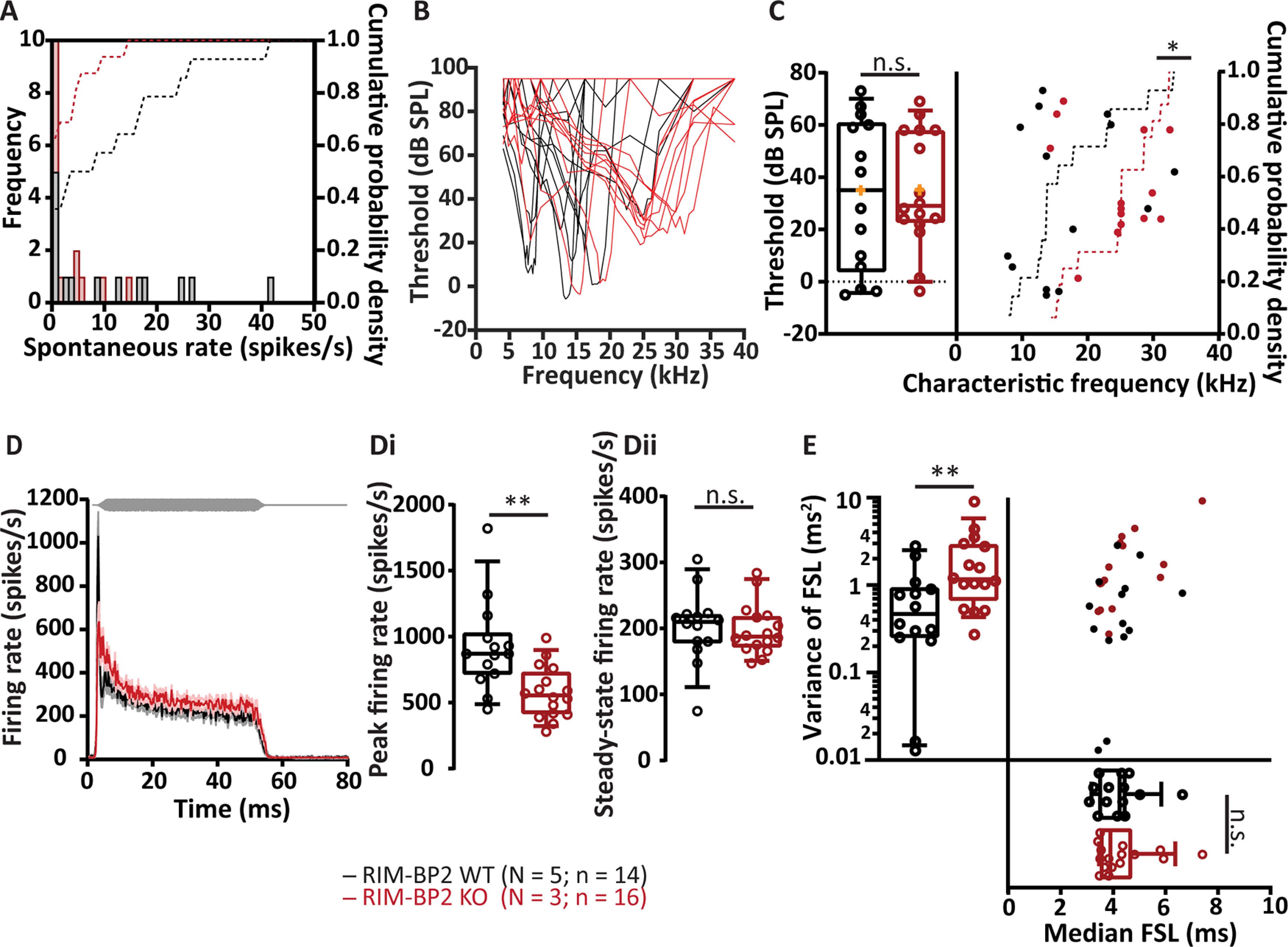
Impaired transmission of sound information in aVCN of RIM-BP2 KO mice *in vivo*. ***A***, Comparable distribution of spontaneous firing rates of single BCs in RIM-BP2 WT (black) and RIM-BP2 KO (red). The histograms represent the frequency distribution (left *y*-axis) and the dotted lines represent the cumulative probability density (right *y*-axis) of spontaneous firing rates. ***B***, ***C***, Representative tuning curves of BCs from RIM-BP2 WT (black) and RIM-BP2 KO (red) demonstrate preserved sharp frequency tuning and low thresholds at the characteristic frequencies (frequency for which spike rate increase requires least sound intensity; ***C***, left) in RIM-BP2 KO BCs. For unknown reasons, we encountered more BCs with high characteristic frequency in RIM-BP2 KO (***C***, right). ***D***, Rise-aligned PSTH of the BC response to 50-ms tone burst stimulation (at characteristic frequency, 30 dB above threshold, stimulus represented in gray) in RIM-BP2 WT (black; *N* = 5; *n* = 14) and RIM-BP2 KO (red; *N* = 3; *n* = 16). PSTH presented as mean (solid lines) ± SEM (shaded area). Peak onset firing rate was significantly reduced in RIM-BP2 KO BCs (***Di***) while the steady-state firing rate was comparable between the two genotypes (***Dii***). Variance in the first spike latency of PSTH (in ***D***) was increased in RIM-BP2 KO units while the median first spike latency remained unperturbed (***E***). Data information: Significance levels: n.s. *p* ≥ 0.05, *p < 0.05, **p < 0.01; *n* = number of BCs; and *N* = number of mice. Box and whisker plot represents median, lower/upper quartiles and 10–90th percentiles. Each data point represents the response of a BC. For details about mean ± SEM, median, sample size, and statistics, see Table 3.

Next, we evaluated the impact of the impaired synaptic transmission on processing of auditory information using juxtacellular recordings from putative spherical BCs (for simplicity referred to as “BCs”) *in vivo* (Fig. 8). Glass microelectrodes were stereotactically navigated to the aVCN from an occipital craniotomy, sound stimuli were presented in the open field, and BCs identified based on electrode position, first spike latency, regularity of firing and shape of the peristimulus time histogram (see Materials and Methods). The (non-significant) trend toward lower spontaneous firing rate (Fig. 8*A*; Table 3) is consistent with the reduced spontaneous ANF input (Krinner et al., 2017) and the trend toward a lower frequency of sEPSCs in RIM-BP2 KO BCs (Fig. 3). Sound threshold and quality of frequency tuning were unaltered, whereby the RIM-BP2 KO data set contained more BCs with higher characteristic frequency (Fig. 8*B*,*C*; Table 3).

**Table 3.** Analysis of *in vivo* extracellular recordings from single BCs

Parameter	RIM-BP2 WT (*N* = 5; *n* = 14)	RIM-BP2 KO (*N* = 3; *n* = 16)	*p* value
Spontaneous rate (spikes/s)	10.90 ± 3.42 (5.88)	2.74 ± 1.07 (0.60)	0.18, M
Threshold (dB SPL)	33.20 ± 7.76 (35.00)	35.20 ± 5.50 (29.00)	0.83, T
Characteristic frequency (kHz)	16.75 ± 2.06 (13.66)	24.21 ± 1.59 (25.05)	**0.015, K**
Peak firing rate (spikes/s)	922.10 ± 91.65 (870.00)	575.60 ± 49.24 (555.00)	**0.0033, TW**
Steady-state firing rate (spikes/s)	202.30 ± 14.53 (209.80)	197.80 ± 9.6 (187.40)	0.79, T
Variance of FSL (ms^2^)	0.76 ± 0.22 (0.47)	2.11 ± 0.56 (1.18)	**0.008, M**
Median FSL (ms)	4.20 ± 0.24 (4.25)	4.36 ± 0.28 (3.90)	0.93, M

Data are presented as mean ± SEM, and medians are shown in parentheses. Normality of data distribution was tested with Kolmogorov–Smirnov test, and the variances were compared with *F* test. Statistical test used to assess the significance of differences is indicated in the column of *p* value. Unpaired Student's *t* test (shown in the table as T) was used, when the data satisfied the criteria of normality and variance comparability. Normally distributed data with unequal variances were compared using Student's *t* test with Welch's correction (shown in the table as TW). Non-normally distributed data were tested with Mann–Whitney *U* test or Kolmogorov–Smirnov test (shown in the table as M and K, respectively); *p* < 0.05, set as threshold for statistical significance. Statistically significant differences are shown in bold.

The peak firing rate at sound onset was significantly reduced in RIM-BP2 KO BCs (Fig. 8*D, Di*, by ∼40%; Table 3) compatible with the reduced initial release probability. The adapted firing rates were not significantly reduced (Fig. 8*D, Dii*; Table 3), which is consistent with the better maintained EPSC amplitudes during steady state response to train stimulation in BCs (RIM-BP2 WT: 1.2 ± 0.2 nA, RIM-BP2 KO: 0.9 ± 0.1 nA; Fig. 5*A–F*; Table 2) and the normal adapted firing rate of ANFs (Krinner et al., 2017).

In addition, we found the temporal jitter of the first spike after stimulus onset to be greater in RIM-BP2 KO BCs, while the first spike latency was comparable between the two genotypes (Fig. 8*E*; Table 3). The stronger reduction of the peak rate and increased first spike latency jitter of BCs in comparison to ANF likely reflects the impaired transmission at the RIM-BP2-deficient endbulb, which degrades information processing in the lower auditory pathway beyond what is caused by the mildly affected synaptic sound encoding in the cochlea. This hypothesis is further supported by the auditory brainstem responses of RIM-BP2 KO mice, which show a more pronounced amplitude decline for the aVCN related Wave III than for the auditory nerve related Wave I (Krinner et al., 2017). In conclusion, the reduced release probability of the endbulbs impairs the transmission of sound onset information, which is likely to hamper hearing and auditory tasks such as gap detection and sound localization in particular.

### RIM-BP2 disruption appears not to alter the molecular composition of the endbulb AZs

**Figure 9.**
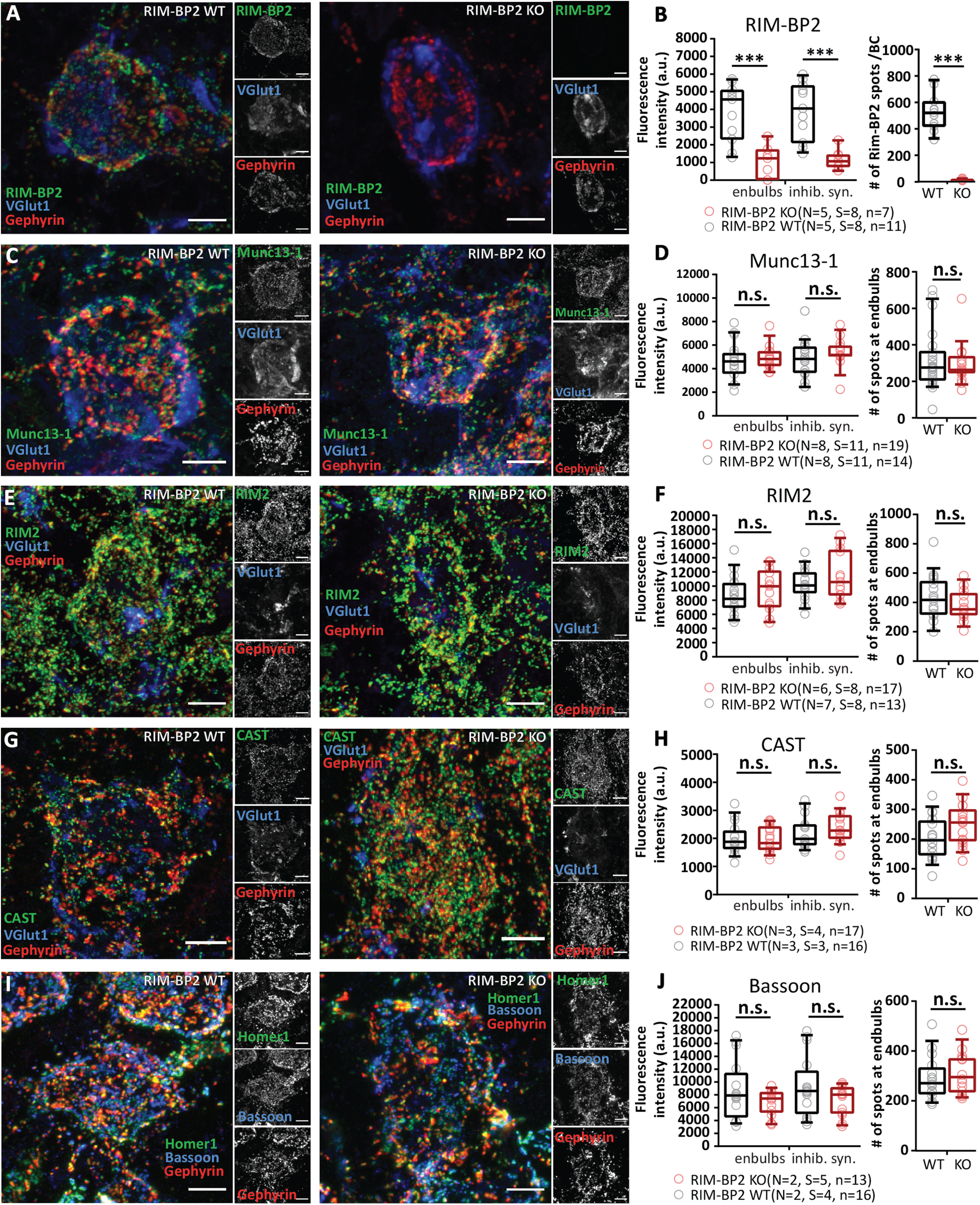
RIM-BP2 disruption does not seem to alter the molecular composition of the endbulb AZs. ***A*, *C*, *E*, *G*, *I***, Representative maximal *z*-projections of confocal image stacks of BCs in RIM-BP2 WT shown on left and RIM-BP2 KO on right; 30-μm coronal brainstem slices were immunolabelled for RIM-BP2 (***A***), Munc13-1 (***C***), RIM2 (***E***), and CAST (***G***) and co-stained for VGlut1 to outline endbulbs and Gephyrin for inhibitory synapse labeling. For Bsn immunolabeling (***I***), we co-stained for Homer1 (excitatory postsynapses) and Gephyrin. ***B*, *D*, *F*, *H*, *J***, Quantification of fluorescence intensity of CAZ proteins at endbulbs and inhibitory synapses of BCs (left), and the number of spots of the target CAZ protein at endbulbs (right). ***A***, Representative *z*-projection shows loss of RIM-BP2 immunoreactivity in the RIM-BP2 KO brainstem slices. There was only a faint residual and likely unspecific signal remaining at excitatory and inhibitory synapses (***B***, left), RIM-BP2 immunofluorescent spots were nearly abolished (***B***, right). The immunofluorescence intensity of Munc13-1 (***D***), RIM2 (***F***), CAST (***H***), and Bsn (***J***) were unaltered at RIM-BP2 KO endbulbs. Right columns of ***D***, ***F***, ***H***, ***J*** show similar number of CAZ protein spots localized at KO endbulb AZs as in the WT AZs. The numbers were calculated by subtracting the Gephyrin colocalized spots from the total number of spots per BC. The data are presented as box and whiskers plots (grand median of mean estimates for all BCs, lower/upper quartiles, 10–90th percentiles). Each data point represents the mean estimate of fluorescence intensity of all the AZs of each BC included in the analysis. Statistical significance of differences between groups was determined with unpaired Student's *t* test (with Welch's correction, when variances differed significantly), if the data's distribution did not differ from a normal distribution or with Mann–Whitney *U* test in case of non-normally distributed data. Normality of distribution was tested with Jarque–Bera test, and variances were compared with *F* test; *p ≤ 0.05, **p ≤ 0.01, ***p ≤ 0.001, n.s. *p* ≥ 0.05. Samples from RIM-BP2 WT and RIM-BP2 KO mice, aged p15–p21, were harvested and processed strictly in parallel, and images were acquired in parallel using the same laser power and gain settings at the same confocal microscope. Data information: *N*: number of animals, *S*: number of slices, *n*: number of BCs. All scale bars: 5 μm.

Given the scope of protein interactions of RIM-BP that includes Ca^2+^ channels, large conductance Ca^2+^ activated K^+^ channels (Sclip et al., 2018) and multidomain proteins of the AZ, we considered the possibility that some of the above described physiological alterations might reflect changes in the abundance of other AZ proteins. In order to test for potential effects of RIM-BP2 disruption on the molecular composition of the AZs, we performed semi-quantitative, confocal immunofluorescence microscopy in coronal brain slices. RIM-BP2 WT and RIM-BP2 KO samples were harvested and processed strictly in parallel. Likewise, images were acquired using the same laser power and gain settings at the same confocal microscope. Excitatory AZs facing the postsynaptic BC were identified by co-localization of immunofluorescence of the targeted AZ protein with the immunofluorescence of the vesicular glutamate transporter VGlut1 or a juxtaposition to the immunofluorescence of Homer 1, a scaffold of excitatory synapses and a lack of juxtaposition to immunofluorescence of Gephyrin, a scaffold of inhibitory synapses (Fig. 9). We focused our analysis on the spherical or ovoid BC soma which are engaged by a corona of synapses. Staining for RIM-BP2 showed the expected corona of immunofluorescence spots in WT slices, but no obvious synaptic immunofluorescence in RIM-BP2 KO slices (Fig. 9*A*,*B*, left: integrated fluorescence within the Vglut1-positive volume, endbulbs: *p* = 0.0008, inhibitory synapses: *p* = 0.0002, right: lack of RIM-BP2 spots in the KO *p* < 0.0001, Student's *t* test). We did not observe significant differences in the integrated immunofluorescence or the number of puncta of Munc13-1 (Fig. 9*C*,*D*), RIM2 (Fig. 9*E*,*F*), CAST (Fig. 9*G*,*H*), and Bsn (Fig. 9*I*,*J*) in RIM-BP2 KO slices (*p* ≥ 0.05), suggesting an unaltered abundance of these multidomain proteins at the excitatory AZs facing the BC in the absence of RIM-BP2.

### Increased Ca^2+^ influx improves release probability but unmasks impaired SV replenishment during train stimulation

**Figure 10.**
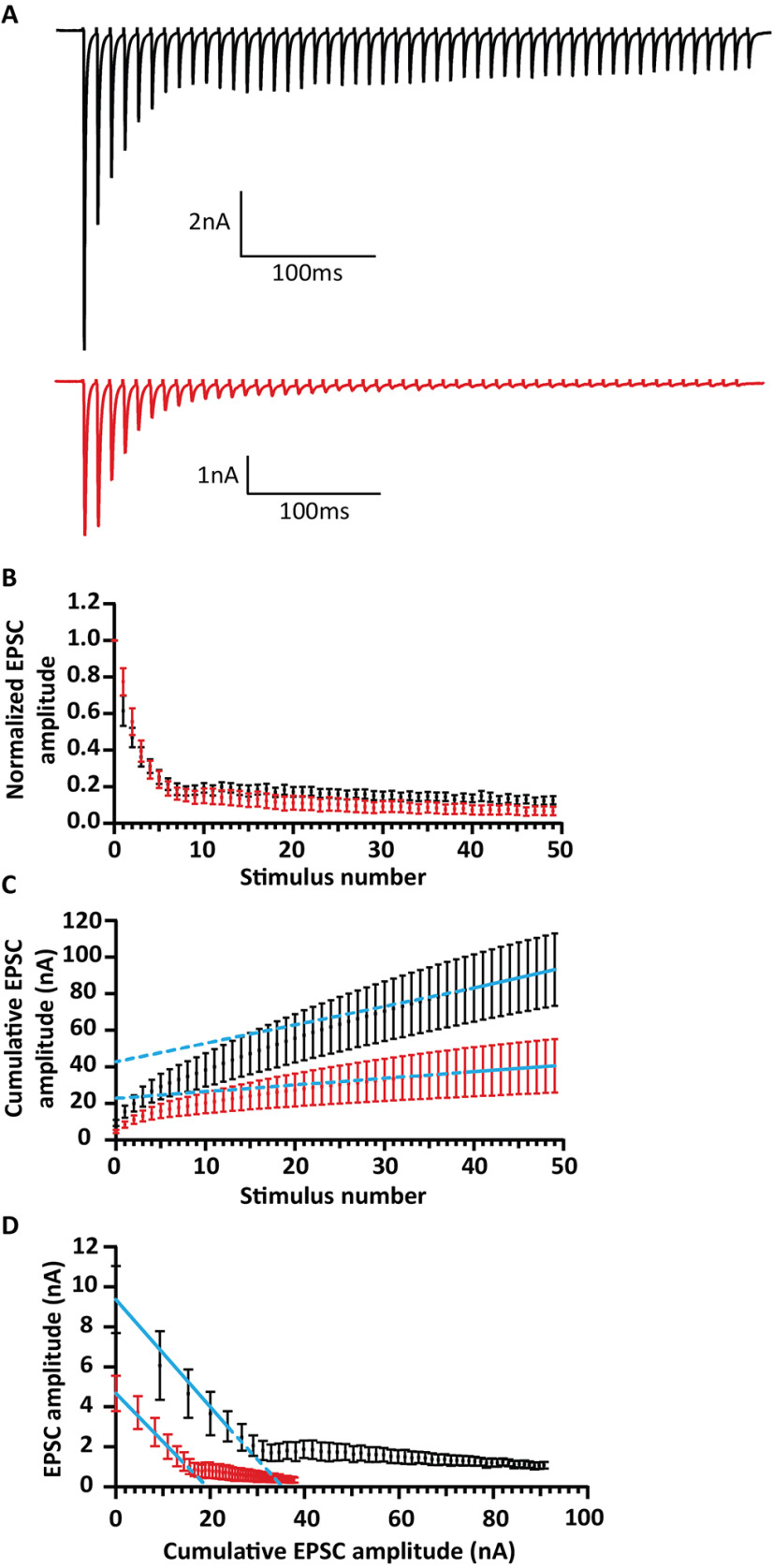
Analysis of release probability (P_r_) as well as the size and dynamics of the RRP at 4 mm [Ca^2+^]_e_. ***A***, Representative traces of eEPSCs in response to trains of 50 action potentials delivered at a 100-Hz frequency, recorded from WT (top, black traces) and RIM-BP2 KO (bottom, red traces). When the mutant terminals are exposed to 4 mm [Ca^2+^]_e_, a WT-like depression pattern is restored. ***B***, This is even more obvious when the mean EPSC amplitudes, normalized to the first EPSC of the train are plotted against the stimulus number. ***C***, We estimate the size of the RRP, the rate of vesicle replenishment during the train and the P_r_ using the SMN method. ***D***, We estimate the RRP size and P_r_ using the EQ method. For 100 Hz: RIM-BP2 WT *N* = 7; *n* = 7, RIM-BP2 KO *N* = 5; *n* = 5. *N*: number of animals; *n*: number of BCs. Quantitative analysis is further elaborated in Table 4.

Unaltered Ca^2+^ influx and RRP size but reduced P_r_ led us to focus the analysis on the coupling of Ca^2+^ channels to SV release. As a first approach we increased the presynaptic Ca^2+^ influx by elevating the extracellular Ca^2+^ concentration [Ca^2+^]_e_ from physiological (2 mm) to 4 mm. This manipulation abolished the differences in P_r_ (both time course of depression and PPR were WT-like; Fig. 10; Table 4). This is consistent with a greater diffusional distance between Ca^2+^ channels and SV release sites that can be overcome when more Ca^2+^ enters per channel opening. Alternatively, or in addition, greater Ca^2+^ influx might foster Ca^2+^-dependent priming or facilitation of release. Finally, increased Ca^2+^ influx unmasked a slowed SV replenishment during train stimulation in RIM-BP2-deficient endbulbs (Fig. 10; Table 4).

**Table 4.** Analysis of P_r_ as well as the size and dynamics of the RRP at 4 mm [Ca^2+^]_e_

Stimulation frequency	Parameter	RIM-BP2 WT	RIM-BP2 KO	*p* value
100 Hz	eEPSC1 amplitude (nA)	9.36 ± 1.67 (8.07)	4.67 ± 0.88 (4.05)	**0.035, T**
	tau (ms)	21.76 ± 3.44 (20.17)	29.19 ± 4.50 (32.89)	0.2115, Τ
	eEPSC_30–50_	1.22 ± 0.21 (1.23)	0.41 ± 0.18 (0.46)	0.2020, M
	eEPSC_30–50_/EPSC_max_	0.14 ± 0.02 (0.13)	0.08 ± 0.03 (0.10)	0.1125, T
	Replenishment	1.91 ± 0.29 (1.96)	0.66 ± 0.26 (0.78)	**0.0116, Τ**
	RRP_SMN_ (SVs)	666.43 ± 219.15 (363.98)	415.15 ± 155.94 (331.33)	0.5303, Μ
	RRP_EQ_ (SVs)	602.61 ± 168.87 (518.41)	358.88 ± 102.66 (320.26)	0.3434, Μ
	P_r SMN_	0.31 ± 0.04 (0.27)	0.26 ± 0.04 (0.22)	0.4168, Τ
	P_r EQ_	0.30 ± 0.04 (0.30)	0.27 ± 0.04 (0.24)	0.6034, Τ
	PPR	0.62 ± 0.08 (0.63)	0.77 ± 0.07 (0.79)	0.2677, Μ

Tau (τ): time constant of single exponential fit to the decay of eEPSC amplitudes during the stimulation train. eEPSC_30–50_/EPSC_max_: average amplitude of the last 20 EPSCs (30–50) in the train, normalized to the amplitude of the largest EPSC of the train. Replenishment: rate of vesicle replenishment during the train. RRP: readily releasable pool. P_r_: release probability. PPR: paired pulse ratio, amplitude of the second EPSC of the train normalized to the amplitude of the first EPSC. Data are presented as mean ± SEM, and medians are shown in parentheses. Normality of data distribution was tested with Jarque–Bera test, and the variances were compared with *F* test. Statistical significance of differences was assessed with unpaired Student's *t* test (shown in the table as T), when the data satisfied the criteria of normality and variance comparability. When the data did not satisfy these criteria, the Mann–Whitney *U* test was used instead (shown in the table as M); *p* < 0.05, set as threshold for statistical significance. Statistically significant differences are shown in bold.

### RIM-BP2 disruption alters the topography of Ca^2+^ channels at endbulb AZs

**Figure 11.**
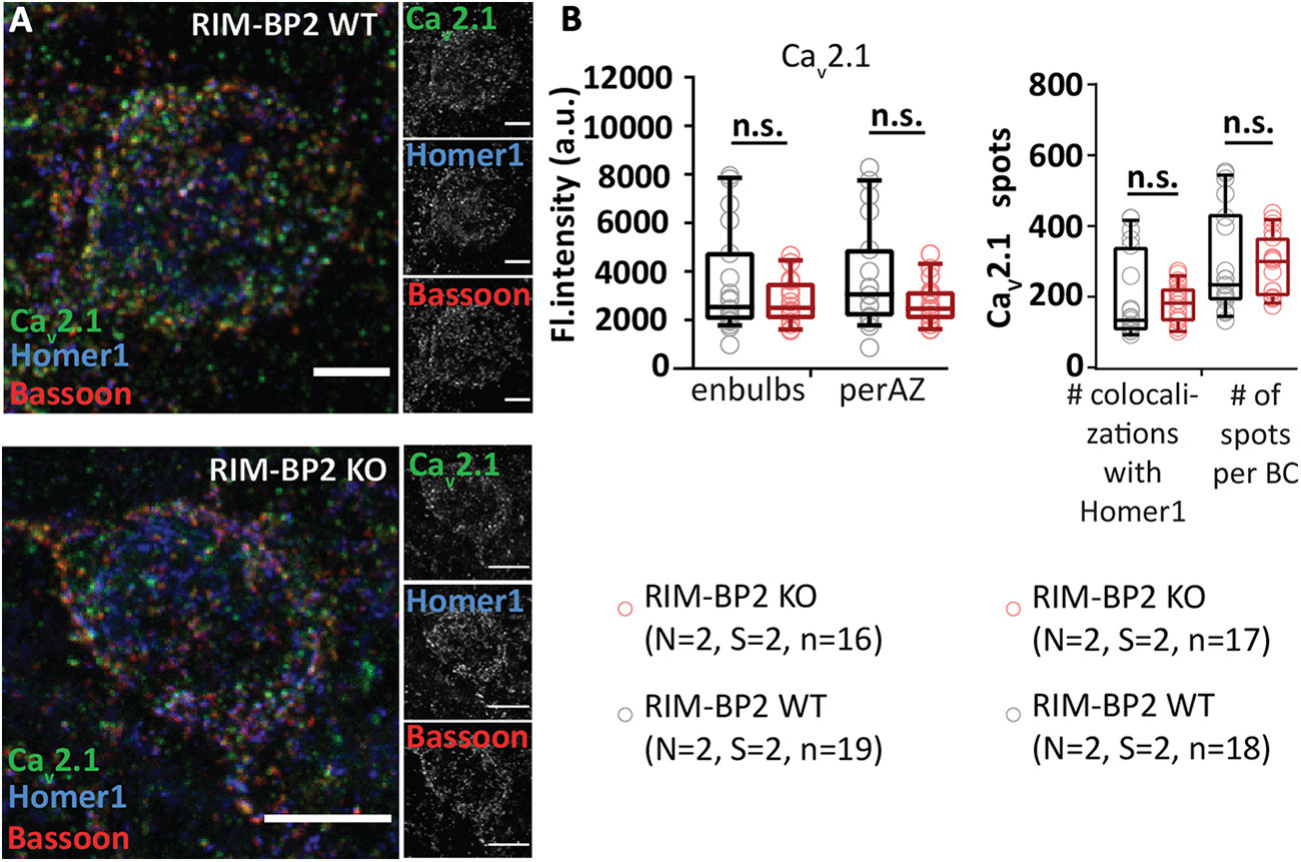
Unaltered Ca_V_2.1 immunofluorescence intensity in RIM-BP2-deficient endbulbs of Held. ***A***, Maximal *z*-projections from confocal image stacks display the immunolabeling against Ca_V_2.1, Bsn, and Homer1 at endbulb AZs in coronal brain stem slices of mouse aVCN. ***B***, No quantitative change was found in the integrated immunofluorescence (left) or number (right) of Ca_V_2.1 puncta in either the excitatory endbulb AZs (juxtaposed to Homer1 immunofluorescence) or all AZs (endbulb + inhibitory AZ) facing BC of RIM-BP2 KOs. n.s. *p* ≥ 0.05. *N*: number of animals, *S*: number of slices, *n*: number of BCs.

**Figure 12.**
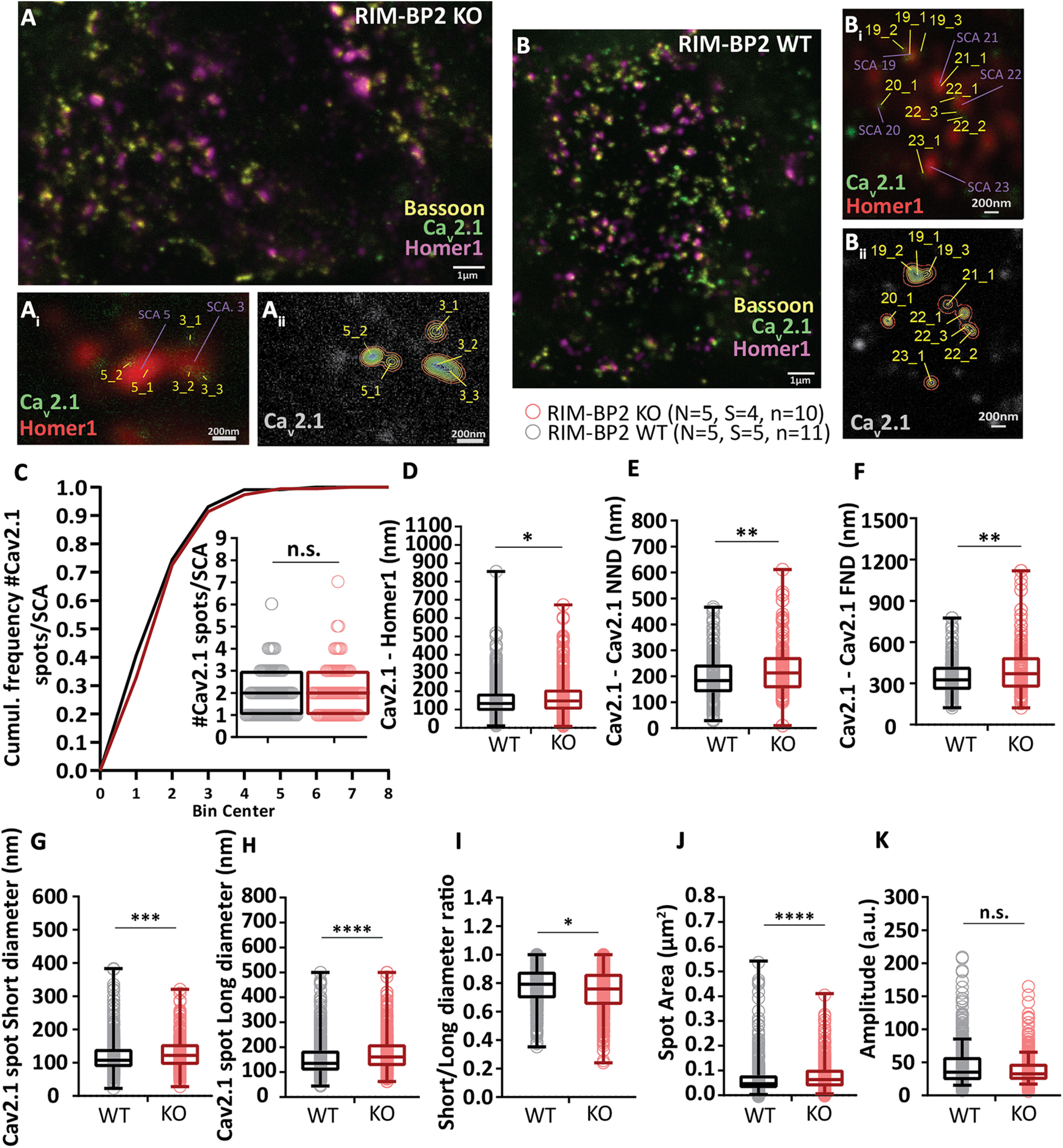
RIM-BP2 disruption alters the topography of Ca^2+^ channels at endbulb AZs. ***A***, ***B***, Analyzing sections from 20-µm-thick brain slices stained for Ca_V_2.1 (STED), Bsn (STED), and Homer1 (Confocal) with STED nanoscopy, uncovered differences in the topography and dimensions of Ca_V_2.1 clusters at AZ–to–PSD SCAs of RIM-BP2 WT (***A*, *A_i_*, *A_ii_***) and RIM-BP2 KO (***B***, ***B_i_***, ***B_ii_***) endbulb terminals. ***C***, The number of clusters per SCA is unchanged in the KO. In the absence of RIM-BP2, the Ca_V_2.1 puncta (yellow tags ***A_i_***, ***A_ii_***, ***B_i_***, ***B_ii_***) are located further from the center of the SCA (***D***), defined as the center of the Ηomer1 puncta (violet tags ***A_i_***, ***B_i_***). The clusters are also located further apart from each other, shown by increased NND (***E***) and FND (***F***) within SCAs. ***G***, ***H***, ***J***, 2D–Gaussian fitting yielded the short and long cluster diameters at half maximum. ***I***, The deletion of RIM-BP2 leads to larger and more elongated Ca_V_2.1 puncta at the endbulb of Held AZs. ***K***, The amplitude of fluorescence at the center of Ca_V_2.1 puncta does not differ significantly between RIM-BP2 WT and RIM-BP2 KO AZs. A semiquantitative assessment of Ca_V_2.1 abundance in endbulb is depicted in Figure 11. The lack of RIM-BP2 also affects the precise localization of the presynaptic protein bassoon. A similar STED–2D Gaussian fitting analysis was employed to dissect the effect on bassoon and the results are summarized in Figure 13. Finally, a comparison of the NND between bassoon and Ca_V_2.1 puncta within SCAs is depicted in Figure 14. Statistical significance between groups was tested with Mann–Whitney *U* test for non-normally distributed data or with Student's *t* test;for normally distributed data with equal variance. ****p < 0.0001, ***p < 0.001, **p < 0.01, *p < 0.05. *N*: number of animals, *S*: number of slices, *n*: number of BCs. The effect sizes and statistics of the performed comparisons are summarized in Table 5.

In order to probe for potential morphologic correlates of altered release probability we employed immunofluorescence microscopy and electron microscopy of immunolabeled Ca^2+^ channels. First, we performed a strictly parallel study of Ca_V_2.1 distribution at excitatory synapses around the BCs of RIM-BP2 KO and WT mice using confocal microscopy (as described above; Fig. 11*A*,*B*) as well as STED nanoscopy (Fig. 12*A-K*). We used Bsn and Homer1 as context markers to analyze the presynaptic Ca_V_2.1 immunofluorescence intensity at endbulbs and at all BC facing AZs, including inhibitory ones. The integrated fluorescence intensity was comparable at both endbulbs (*p* = 0.65, Mann–Whitney *U* test; Fig. 11*B*), and at all BC facing AZs (*p* = 0.11, Student's *t* test; Fig. 11*B*). The number of Ca_V_2.1 spots neighboring Homer1 appositions (*p* = 0.60, Student's *t* test; Fig. 11*B*) and total number of spots facing BC (*p* = 0.87, Student's *t* test; Fig. 11*B*) were unaltered at the confocal level, which is consistent with finding normal presynaptic Ca^2+^ influx (Fig. 4).

**Table 5.** Altered Ca_V_2.1 shape properties and spatial distribution in endbulb SCAs

	RIM-BP WT VGCCs	RIM-BP KO VGCCs	*p* value
Amplitude	44.77 ± 1.54 (35.14)	38.55 ± 1.16 (32.55)	n.s. 0.1061, M
Short diameter	120.39 ± 2.47 nm (106.98)	127.6 ± 2.39 nm (121.98)	***0.0005, M
Long diameter	158.97 ± 3.58 nm (135.58)	178.72 ± 3.91 nm (160.98)	****<0.0001, M
Area (μm^2^)	0.071 ± 0.004 μm^2^ (0.047)	0.079 ± 0.003 μm^2^ (0.064)	****<0.0001, M
Ratio	0.78 ± 0.007 (0.79)	0.74 ± 0.008 (0.76)	**0.0019, M
Distance from left (nm)	151.47 ± 4.3 nm (133.12)	167.84 ± 5.45 nm (146.71)	*0.0399, M
NND (nm)	196.25 ± 6.3 nm (183.78)	227.6 ± 7.58 nm (212.72)	**0.0032, M
FND (nm)	347 ± 9.4 nm (325.27)	411.43 ± 14.87 nm (370.03)	**0.0037, M

Data are presented as mean ± SEM (median). Statistical significance of differences was assessed with unpaired Student's *t* test (shown in the table as T), when the data satisfied the criteria of normality and variance comparability. When the data did not satisfy these criteria, the Mann–Whitney *U* test was used instead (shown in the table as M); *p* < 0.05, set as threshold for statistical significance. Statistically significant differences are shown in bold;

****p < 0.0001,

***p < 0.001,

**p < 0.01,

*p < 0.05.

**Figure 13.**
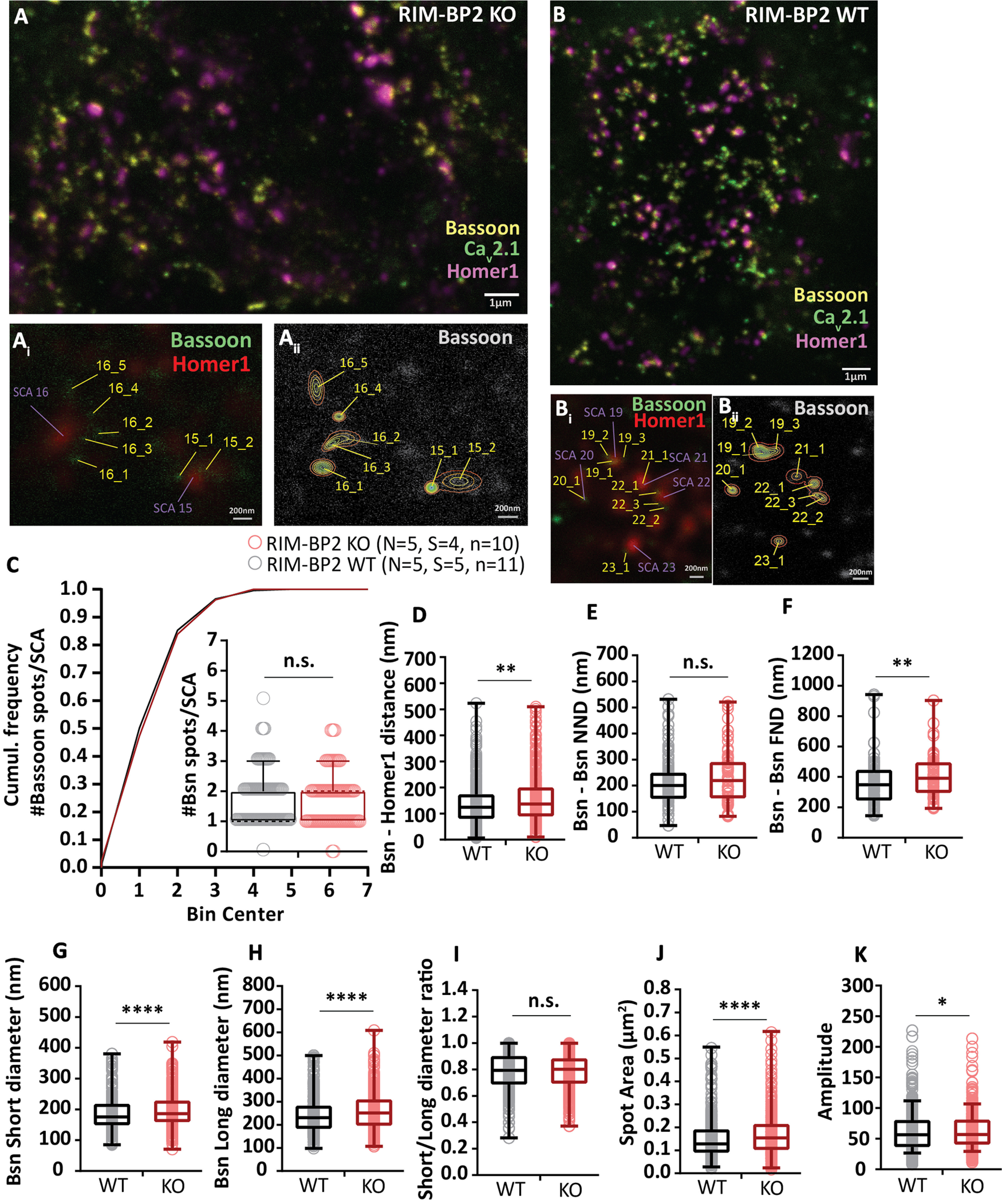
RIM-BP2 disruption alters distribution and extent of Bsn clusters marking the presynaptic density of endbulb AZs. Analyzing sections (***A***, ***B***) from 20-µm-thick brain slices stained for Ca_V_2.1 (STED), Bsn (STED), and Homer1 (confocal) with STED nanoscopy, uncovered differences in the amplitude of fluorescence, topography, and dimensions of Bsn-positive puncta in the absence of RIM-BP2. The Bsn puncta were detected and fitted with 2D–Gaussian functions at AZ–to–PSD SCAs of RIM-BP2 WT (***A***, ***A_i_***, ***A_ii_***) and RIM-BP2 KO (***B***, ***B_i_***, ***B_ii_***) endbulb terminals. The number of puncta per SCA is unchanged in the KO (***C***). In the absence of RIM-BP2, the Bsn puncta (yellow tags ***A_i_***, ***A_ii_***, ***B_i_***, ***B_ii_***) are located further from the center of the SCA (***D***), defined as the center of the postsynaptic Homer1 puncta (violet tags ***A_i_***, ***B_i_***). We found no significant difference in the NND (***E***). FND (***F***) was significantly increased in the mutant. Comparing the short and long puncta diameters at half maxima shows that the deletion of RIM-BP2 leads to proportionally (no change in short/long diameter ratio, ***I***) larger (***G***, ***H***, ***J***) Bsn puncta at endbulb of Held AZs. The amplitude of fluorescence at the center of Bsn puncta is significantly increased RIM-BP2 KO AZs (***K***). Normality was tested with Jarque–Bera test. Statistical significance between groups was tested with Mann–Whitney *U* test for non-normally distributed data and with Student's *t* test for normally distributed data with equal variance. ****p < 0.0001, ***p < 0.001, **p < 0.01, *p < 0.05. *N*: number of animals, *S*: number of slices, *n*: number of BCs.

For our two-color STED analysis, we focused on AZ/PSD appositions (Ca_V_2.1/Homer1, Bsn/Homer1) of endbulbs of Held. We interpret the Ca_V_2.1 and Bsn puncta discerned by STED to represent individual AZs. As the PSD was studied at confocal resolution, we assume the larger Homer1-spots represent a merger of several small PSDs. We operationally defined the organization of several Ca_V_2.1 or Bsn puncta around a single Homer1 spot a synaptic contact assembly (SCA) whereby its center corresponds to the center of the Homer1 punctum. The number of Ca_V_2.1 puncta per SCA was comparable between RIM-BP2 WT and KO endbulbs (Fig. 12*C*). However, the Cav2.1 puncta were located further from the center of the SCA (Fig. 12*D*). The distance between Ca_V_2.1 puncta was increased as assessed by NND and furthest neighbor distance (FND) in the SCA (Fig. 12*E*,*F*; Table 5). 2D Gaussian fitting of the Ca_V_2.1 puncta showed that the KO puncta are wider and longer but also more round-shaped than WT (Fig. 12*G–J*), while the fluorescence amplitude at the center of the fitted Ca_V_2.1 puncta was comparable between WT and KO (Fig. 12*K*). Similar findings were made for Bsn when comparing WT and KO SCAs (Fig. 13). Notably, the distance from the SCA center was increased from 138.5 ± 4.6 nm to 160.9 ± 5.7 nm in the KO (*p* = 0.0074, Mann–Whitney *U* test; Fig. 13*D*). The NND between Bsn puncta was not significantly increased in the absence of RIM-BP2 (*p* = 0.156, Mann–Whitney *U* test; Fig. 13*E*), but the FND was altered from 357.5 ± 13.3 nm in WT to 408 ± 15.2 nm in the KO (*p* = 0.0042, Mann–Whitney *U* test; Fig. 13*F*). The Bsn puncta short diameter was increased from 181.7 ± 2.7 nm in the WT to 197.8 ± 3.1 nm in the KO (*p* < 0.0001, Mann–Whitney *U* test; Fig. 13*G*), while the long diameter changed from an average of 239.4 ± 4.3 nm in WT to 261.2 ± 4.6 nm in the KO (*p* < 0.0001, Mann–Whitney *U* test; Fig. 13*H*).In contrast to our Ca_V_2.1 findings, the lack of RIM-BP2 did not affect the Bsn puncta short/long diameter ratio (*p* = 0.6043, Mann–Whitney *U* test; Fig. 13*I*), while the amplitude of Bsn fluorescence was slightly increased in AZs of RIM-BP2 lacking endbulbs [63.9 ± 1.9 (KO) vs 60.3 ± 1.9 (WT), Mann–Whitney *U* test *p* = 0.0363; Fig. 13*K*]. The area of Bsn puncta was increased on average from 0.15 ± 0.005 to 0.17 ± 0.006 µm^2^ (*p* < 0.0001, Mann–Whitney *U* test; Fig. 13*J*) in the absence of RIM-BP2.

**Figure 14.**
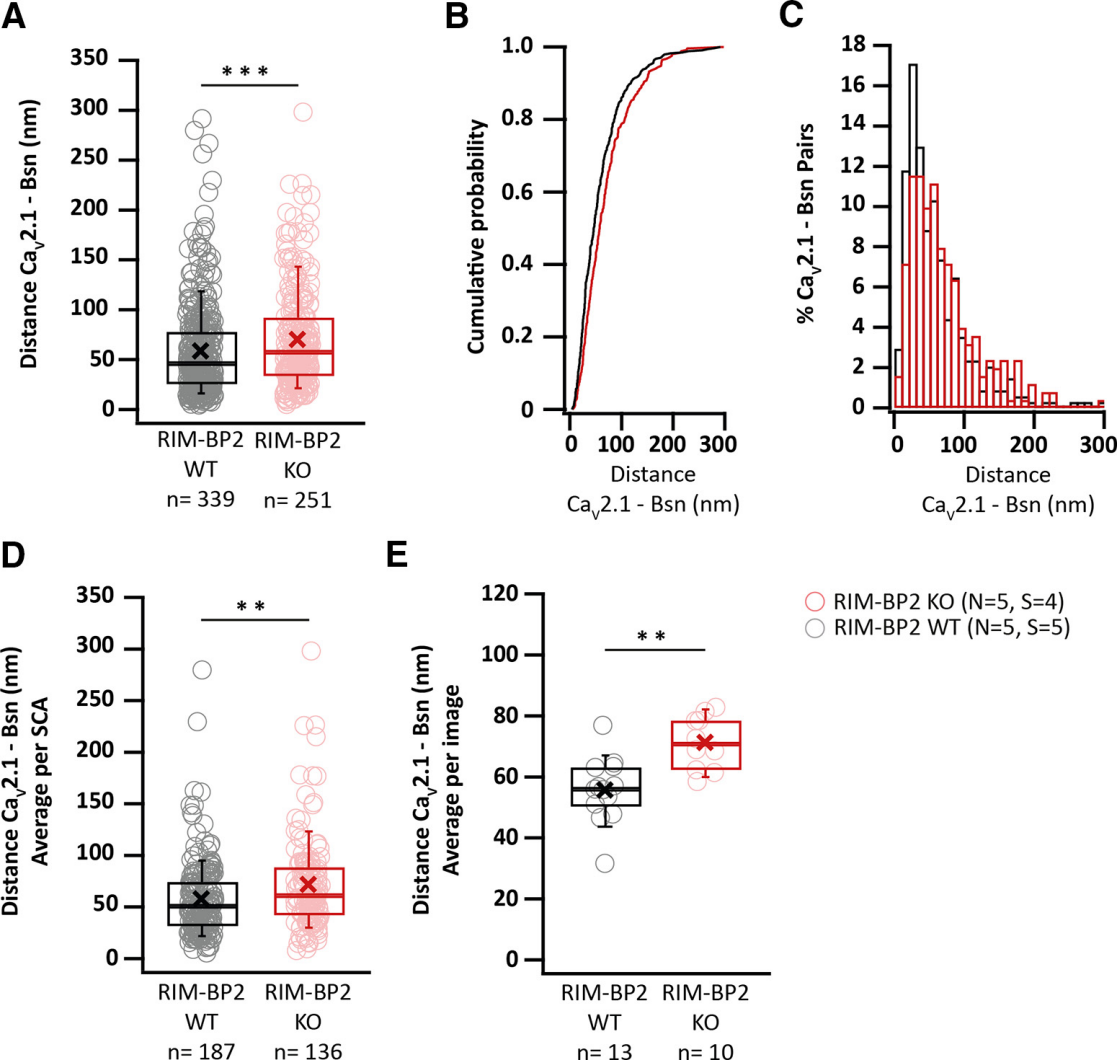
Increased NND between Ca_V_2.1 and Bsn puncta. ***A***, Ca_V_2.1–Bsn, defined at the distance cutoff of 300 nm, show significantly higher NND in the absence of RIM-BP2 compared to WT (WT pairs: *n* = 339, KO pairs: *n* = 251). Cumulative probability (***B***) and histogram (***C***) representations of the NND estimates for Ca_V_2.1–Bsn pairs show that RIM-BP2 KO pairs are less likely to have small NND values compared to WT and more RIM-BP2 KO pairs occupy the higher NND bins. ***D***, The increase in NND is robust and remains statistically significant when averaging the NNDs per-SCA (WT SCAs: *n* = 187, KO SCAs: *n* = 136) and (***E***) also when averaging the NNDs per image taken typically including the SCAs of one BC (WT images: *n* = 13, KO images: *n* = 10) Non-normally distributed data are presented as box and whisker plots with grand median of all BC means (***A***), all SCA means (***D***), and all image means (***E***). Lower/upper quartiles represent the 25/75th percentiles, while the X represents the grand mean (***A***, ***C***, ***D***); n.s. *p* ≥ 0.05; *p ≤ 0.05, **p ≤ 0.01, ***p ≤ 0.001, Mann–Whitney *U* test). *N*: number of animals, *S*: number of imaged slides, *n*: number of pairs (***A***), number of SCAs (***D***), number of images (***E***).

We then assessed the NND between Ca_V_2.1 and Bsn puncta. To calculate the NND we used the x, y coordinates of each punctum's center, yielded from the 2D Gaussian fitting that was applied to analyze the punctum's shape as mentioned above. To define a Ca_V_2.1–Bsn pair, we set a distance cutoff at 300 nm, since <3% of all pairs had NNDs higher than 300 nm. By averaging over all the Ca_V_2.1–Bsn pairs, we found an NND of 58.93 ± 2.6 nm (mean ± *SEM*, *n* = 339) for WT and a significantly higher NND of 70.3 ± 3.1 nm (mean ± SEM, *n* = 251, *p* = 0.0006) in the absence of RIM-BP2 (Fig. 14*A*). Ca_V_2.1–Bsn pairs of RIM-BP2-deficient SCAs were found less frequently than WT ones in the small NND bins, while being more abundant than WT in the higher NND bins (Fig. 14*B*,*C*). The significant increase in NND was robust and persisted when averaging NNDs per SCA (WT mean NND ± SEM = 57.9 ± 2.7 nm, *n* = 187, KO mean NND ± SEM = 71.9 ± 4.5 nm, *n* = 136, *p* = 0.00134; Fig. 14*D*) or per image taken (WT mean NND ± SEM = 55.7 ± 2.9 nm, *n* = 13, KO mean NND ± SEM = 71.3 ± 2.8 nm, *p* = 0.00101; Fig. 14*E*). We also assessed the potential contribution of unspecific labeling for Ca_V_2.1 and Bsn in shaping this effect. To that end we defined orphaned Ca_V_2.1 and Bsn puncta in each SCA, as those that were not found in the proximity of Bsn and Ca_V_2.1, respectively, and quantified their abundance per SCA. We found no significant differences in the amount of orphaned Ca_V_2.1 or Bsn puncta between WT and RIM-BP2 KO SCAs (data not shown).

**Figure 15.**
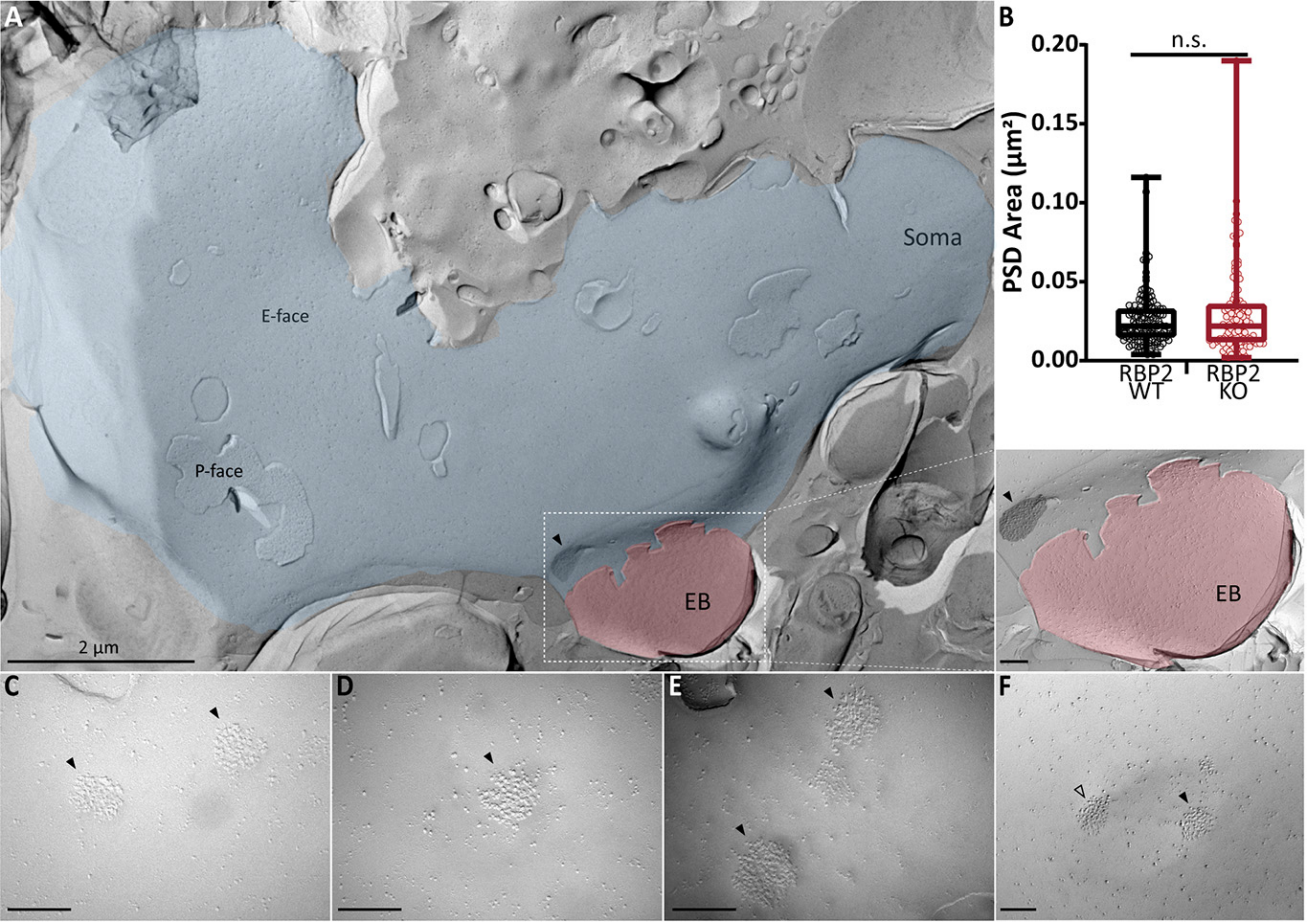
Identification of endbulb of Held synapses. ***A***, SDS-FRIL electron micrograph at low magnification (9700×) showing E-face of a BC soma (blue) with patches of P-face, contacted by an endbulb (red). IMP-cluster representing the PSD of auditory nerve fiber on the BC soma highlighted in black with a solid black arrowhead. Inset shows the magnified (97,000×) view of the endbulb synapse on to the BC with a PSD IMP-cluster. ***B***, Comparable PSD areas at endbulbs of Held in RIM-BP2 WT and KO. ***C–F***, High-magnification (***C–E***, 93,000×; ***F***, 97,000×) images of IMP-clusters of BC soma facing the endbulb. PSD IMP-clusters indicated by solid black arrowheads. Open black arrowhead in ***F*** marks the PSD IMP-cluster that lies on the curvature of the synaptic cleft depression and hence was not included in the analysis. All unmarked scale bars are 200 nm. For details about PSD values, sample size, and statistics, see Table 6.

**Figure 16.**
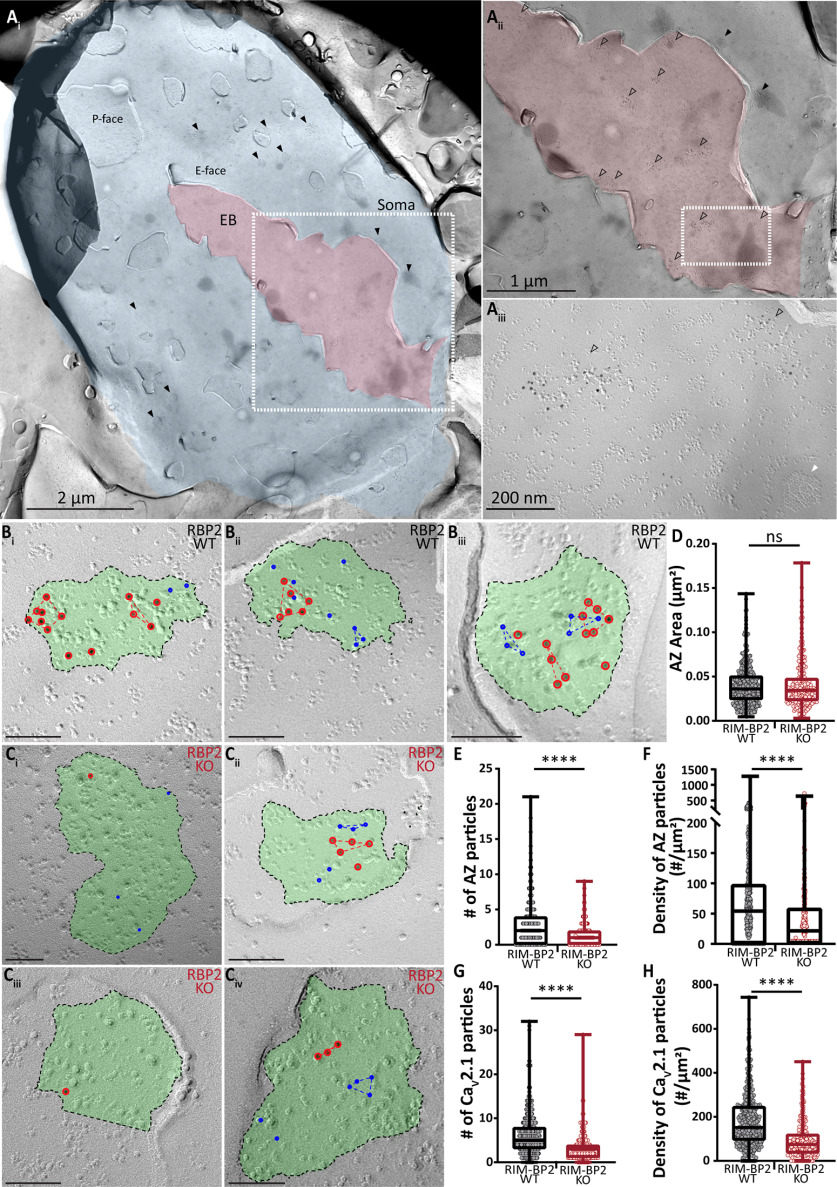
Similar AZ size but reduced number of AZ and Ca_V_2.1 labels in endbulbs of RIM-BP2 KO. ***A***, SDS-FRIL electron micrograph at low magnification (9700×) showing E-face of a BC soma (blue) with patches of P-face, connected to an endbulb (red). ***Ai***, Some of the IMP-clusters representing the PSDs of ANF on the BC soma highlighted by solid black arrowheads. ***Aii***, Magnified (18,500×) view of the box in ***Ai*** of endbulb synapse (red) on to the BC soma (blue) with IMP-clusters for AZ and PSD indicated by open and solid black arrows, respectively. ***Aiii***, Magnified (97,000×) view of the box in ***Aii*** with IMP-clusters for AZ indicated by open arrows and P-face of a gap junction indicated by a solid white arrow. ***B***, ***C***, FRIL images showing endbulb AZs (green) in RIM-BP2 WT (***B***) and KO (***C***), immunolabelled for AZ molecules (RIM, neurexin, ELKS) and Ca_V_2.1 channels with 5-nm (blue circles) and 10-nm (red circles) gold particles, respectively. ***D–H***, Quantitative analysis of AZ area (***D***), number of AZ (***E***), and Ca_V_2.1 (***G***) gold particles, and density of AZ (***F***) and Ca_V_2.1 (***H***) gold particles. All unlabeled scale bars are 100 nm. ns: not significant, ****p < 0.0001. For details about mean ± SEM, median, sample size, and statistics, see Table 6.

We then turned to immunolabeling of Ca_V_2.1 in SDS-treated freeze-fracture replica (SDS-FRIL; Nakamura et al., 2015). To image the endbulbs onto the BCs, we focused on the BC rich rostral-most aVCN sections of the brainstem. Endbulb terminals were prominently distinguishable in our replicas by their large size synapsing on to the BC soma (Fig. 15*A*). We also validated that we were imaging the correct area and cell type, by analyzing the intramembrane particle (IMP) clusters representing PSDs on the exoplasmic face (E-face) of the BC soma (Fig. 15*C–F*). Our estimates of PSD areas (Fig. 15*B*; Table 6) were comparable to the ones previously reported for ANF-BC synapses (Rubio et al., 2017). We then assessed the protoplasmic face (P-face) of the replicas for the analysis of AZ proteins and Ca_V_2.1 channel distribution (Fig. 16). AZs were located by simultaneous immunolabeling of three characteristic AZ proteins: RIM, neurexin, and ELKS with 5-nm gold particles. The number of AZ particles was less than that previously observed in other types of synapses (Miki et al., 2017) which might reflect lower expression of ELKS in the endbulb synapses. Since the samples from both genotypes were handled simultaneously by the same experimenter, the comparison between the genotypes remains valid. Nonetheless, given the low labeling efficiency for AZ proteins, we used AZ markers primarily to identify the location of AZs. AZ area was characterized by IMPs of distinct shape, number and size compared to those in surrounding areas, and demarcated manually by connecting the outermost IMPs (Fig. 16*B*,*C*). The estimated AZ area was comparable between RIM-BP2 KO and WT (Fig. 16*D*). When analyzing the distribution of Ca_V_2.1 channels labeled with 10-nm gold particles within the AZ area, both the number and density of Ca_V_2.1 particles were significantly reduced in the RIM-BP2 KO (Fig. 16*G*,*H*; Table 6).

**Table 6.** Quantitative analysis SDS-FRIL electron micrographs

Au particles		Parameter	RIM-BP2 WT	RIM-BP2 KO	*p* value
		# of animals	3	3	-
		# of replicas	6	5	-
		# of images	437	196	-
		# of PSD	136	102	-
		AZ area (µm^2^)	0.040 ± 0.001 (0.036)	0.041 ± 0.002 (0.034)	0.75, M
		PSD area (µm^2^)	0.026 ± 0.002 (0.022)	0.030 ± 0.003 (0.022)	0.89, M
AZ (5 nm)		# of particles/AZ area	2.74 ± 0.14 (2.00)	1.34 ± 0.12 (1)	**<0.0001, M**
		Density (#/µm^2^)	76.65 ± 4.72 (54.13)	47.91 ± 6.21 (21.41)	**<0.0001, M**
Ca_V_2.1 (10 nm)		# of particles/AZ area	6.30 ± 0.22 (5.00)	3.31 ± 0.23 (3.00)	**<0.0001, M**
		Density (#/µm^2^)	181.10 ± 5.93 (151.30)	96.88 ± 6.19 (73.72)	**<0.0001, M**
Ca_V_2.1 (10 nm)		# of clusters	342	74	-
		# of particles/cluster	4.36 ± 0.13 (4.00)	3.80 ± 0.16 (3.00)	**0.0011, M**
		Area of cluster (nm^2^)	976.25 ± 88.99 (590.90)	659.50 ± 95.37 (418.3)	**0.013, M**
NND analysis					
			95% CI	Model comparison (vs null model)	
		Mean ± SEM	Lower–higher	BF (full/null)	ΔWAIC (null–full)
Ca_V_2.1–Ca_V_2.1	WT	37.8 ± 1.52	33.4–43.8	0.458	−0.2
	KO	47 ± 2.43	40.2–54.6		
AZ–Ca_V_2.1	WT	62.8 ± 7.67	44.6–89.7	0.083	−0.4
	KO	77.4 ± 11.05	51.8–111.3		

Data are presented as mean ± SEM with median in parentheses (except for the NND analysis). Data were distributed non-normally as determined by Kolmogorov–Smirnov test. Statistical significance of the comparison between RIM-BP2 WT, and KO was determined by Mann–Whitney *U* test (denoted in the table as M). *p* < 0.05 set as threshold for statistical significance. Statistically significant differences shown in bold.

**Figure 17.**
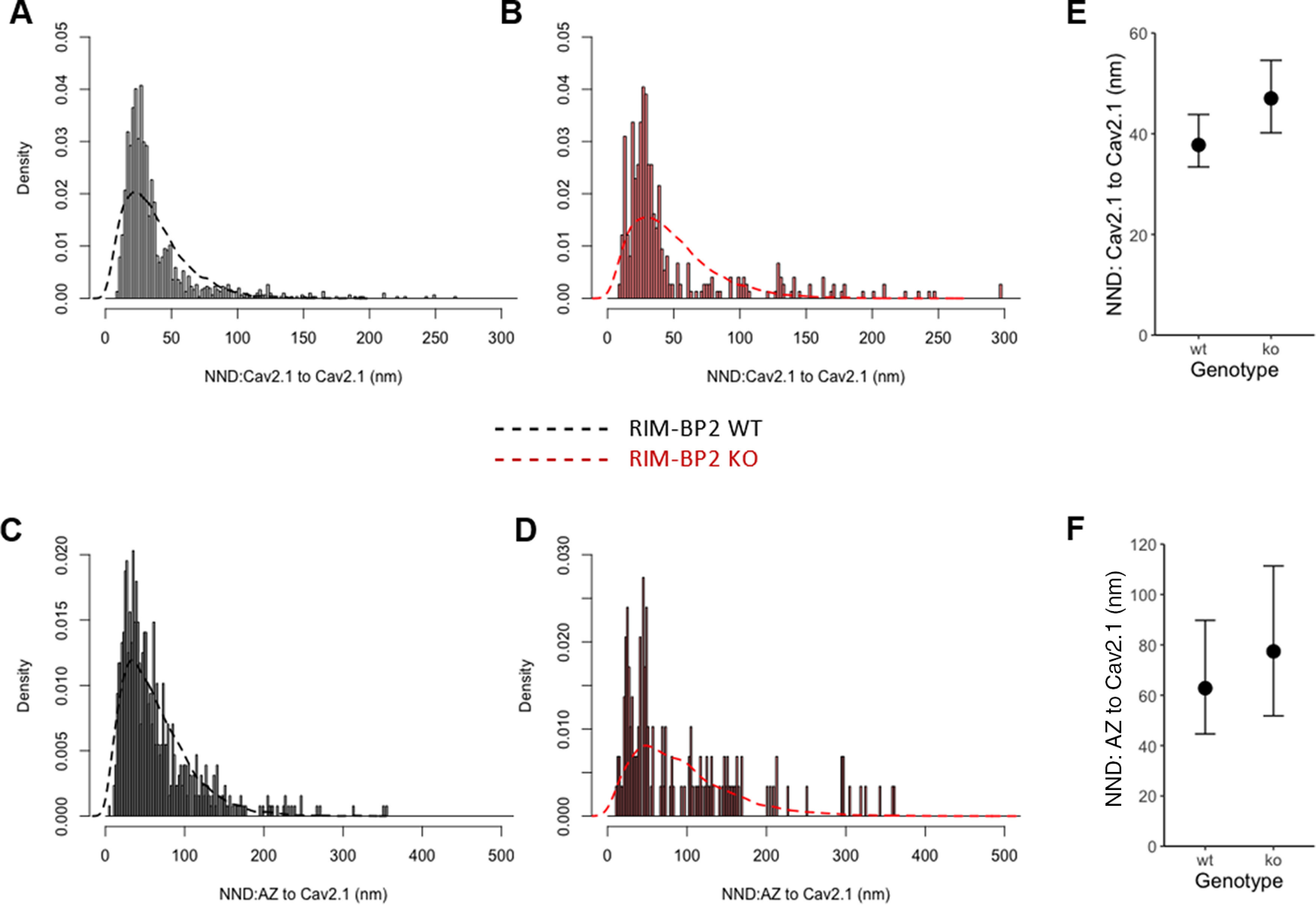
Altered topography of Ca_V_ channels relative to AZ proteins. ***A***, ***B***, Example histograms of NNDs between Ca_V_2.1 to Ca_V_2.1 gold particles in RIM-BP2 WT (***A***) and KO (***B***). ***C***, ***D***, Histogram of NNDs between AZ to Ca_V_2.1 gold particles in RIM-BP2 WT (***C***) and KO (***D***). The dashed lines in (***A–D***) represent kernel density estimators of the predicted NND distributions in each genotype simulated from the hierarchical Bayesian models (see Materials and Methods). ***E***, ***F***, Marginal means of NNDs between Ca_V_2.1 and Ca_V_2.1 (***E***) and AZ-Ca_V_2.1 (***F***) in WT and RIM-BP2 KO animals. Error bars indicate 95% credible intervals (CIs) of the means. For details about the statistics including mean, SEM, 95% CI, and model comparisons, see Table 6. For reference images of endbulbs as seen in the freeze fracture replica, and an analysis of PSD size, see Figure 15. For an assessment of AZ size, and labeling efficiency of AZ and Ca_V_2.1 particles, see Figure 16. Data were from different animals within a genotype and were pooled together for the final analysis. Observed gold particle distributions were compared to 500 random simulations of gold particle distributions to ensure that the reported results did not occur by chance. For a comparison of real and simulated data, see Table 7.

A low labeling efficiency for AZ proteins would overestimate the AZ to AZ and Ca_V_2.1 to AZ particles NNDs. Hence, we only compared the NND for Ca_V_2.1 to Ca_V_2.1 and AZ to Ca_V_2.1 particles. The value of mean ± 2 SD s of the Gaussian fit to the distribution of Ca_V_2.1 to Ca_V_2.1 NNDs was considered the threshold for the maximum distance by which two particles can be separated and still belong to the same cluster (Fig. 16*A*-*C*; see Materials and Methods): mean ± 2 SD were 43.09 and 43.92 nm for WT and RIM-BP2 KO, respectively. The threshold used for defining a cluster in our analysis was 40 nm as used in previous analysis (Miki et al., 2017). In many KO AZ images, there were not even the minimum required three Ca_V_2.1 gold particles to qualify as a cluster (Fig. 16*C*). Within the qualified clusters, we still found a significantly reduced number of Ca_V_2.1 particles per cluster in the RIM-BP2 KO (Table 6) which is consistent with a more dispersed Ca_V_2.1 topography at the AZ as indicated in the STED analysis. Since there were no significant differences between the animals within the groups (RIM-BP2 WT and RIM-BP2 KO; data not shown), data across animals of one genotype were pooled to represent that genotype group. To confirm that the clusters obtained in our analysis were valid and not reflecting chance occurrence, we compared our real data to 500 random simulations of cluster arrangements (see Materials and Methods; Luján et al., 2018; Kleindienst et al., 2020). Table 7 confirms the validity of AZ and Ca_V_2.1 clusters. We then used hierarchical Bayesian modeling of the data to test for differences between the Ca_V_2.1 to Ca_V_2.1 NNDs and AZ to Ca_V_2.1 NNDs of RIM-BP2 WT and RIM-BP2 KO, respectively (Fig. 17). In our limited data sample of AZs from three mice each, we did not observe statistically significant differences for either Ca_V_2.1 to Ca_V_2.1 NNDs (Bayes factor K: 0.46 and Watanabe–Akaike Information Criterion: −0.2; Table 6) or AZ to Ca_V_2.1 NNDs (Bayes factor K: 0.08 and Watanabe–Akaike Information Criterion: −0.4; Table 6). In summary, the major findings of our FRIL analysis are the significantly lower number and density of Ca_V_2.1 channels.

**Table 7.** Comparison of real and simulated distribution of gold particles

		Cluster analysis			
Genotype	Au particles	Parameter	Real data	Simulated data	*p* value
RIM-BP2 WT	AZ (5 nm)	# of clusters/AZ	0.29 ± 0.03	0.14 ± 0.01	**3.68e-16**
		# of particles/cluster	3.99 ± 0.15	3.50 ± 0.09	**8.57e-08**
		Area of cluster (nm^2^)	663.30 ± 80.9	503.78 ± 45.30	**0.007**
	Ca_V_2.1 (10 nm)	# of clusters/AZ	0.78 ± 0.04	0.48 ± 0.03	**6.44e-29**
		# of particles/cluster	4.36 ± 0.13	3.79 ± 0.08	**5.72e-13**
		Area of cluster (nm^2^)	976.25 ± 88.99	695.77 ± 55.90	**2.26e-08**
RIM-BP2 KO	AZ (5 nm)	# of clusters/AZ	0.08 ± 0.02	0.05 ± 0.01	**0.015**
		# of particles/cluster	4.33 ± 0.43	3.71 ± 0.33	**0.006**
		Area of cluster (nm^2^)	759.82 ± 186.11	542.89 ± 125.05	0.09
	Ca_V_2.1 (10 nm)	# of clusters/AZ	0.38 ± 0.04	0.15 ± 0.02	**6.13 e-13**
		# of particles/cluster	3.80 ± 0.16	3.35 ± 0.06	**0.0003**
		Area of cluster (nm^2^)	659.50 ± 95.37	438.90 ± 29.90	**0.005**

Data are presented as mean ± SEM. Real data were compared to 500 random simulations to confirm that the observed clusters are significantly different from chance (random) occurrence. Student's *t* test was used to determine the statistical significance. *p* < 0.05 set as threshold for statistical significance. Statistically significant differences shown in bold.

### RIM-BP2 disruption alters the SV organization at the endbulb AZs

**Figure 18.**
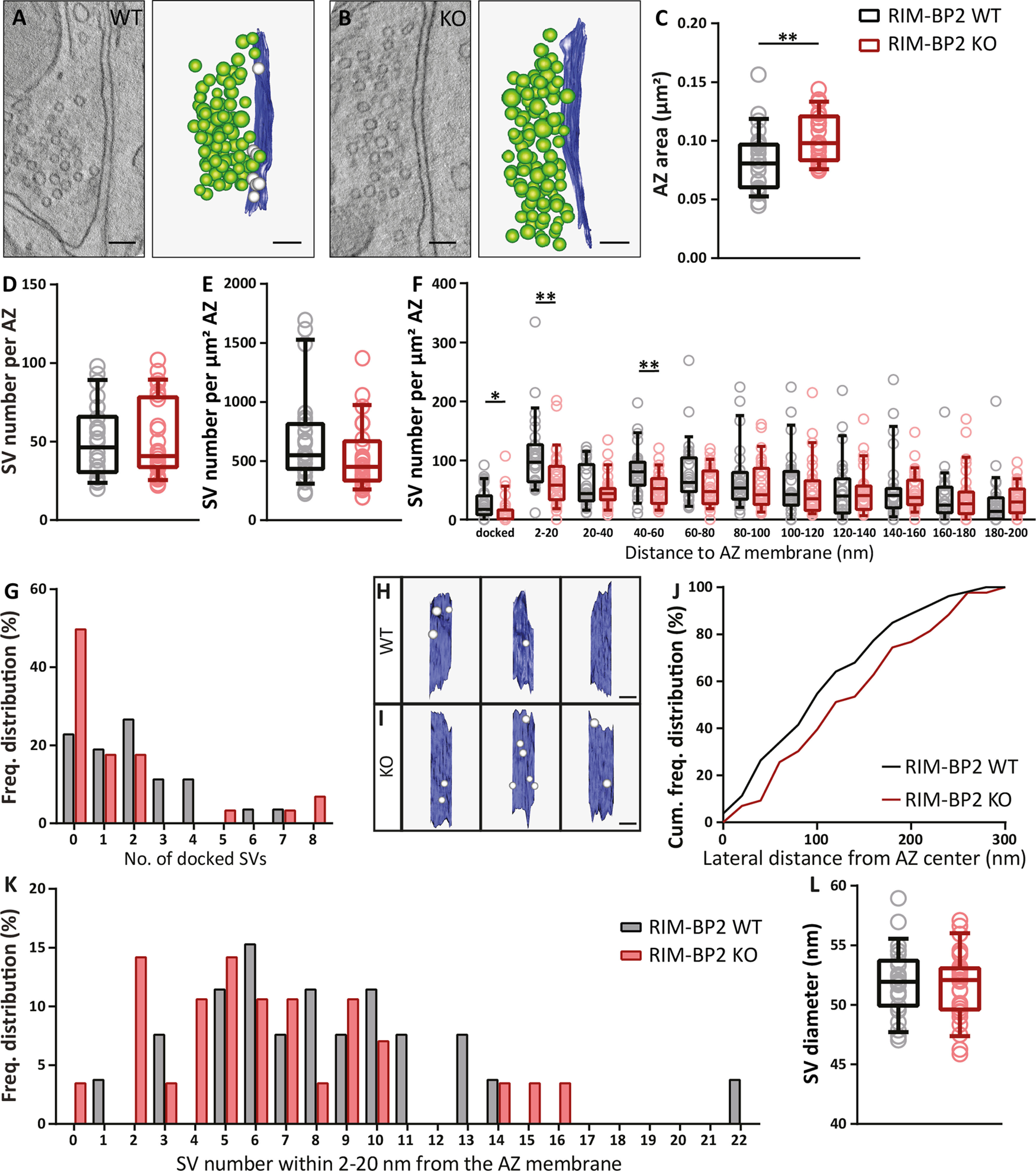
RIM-BP2 disruption alter the axial SV distribution at the endbulb AZs. ***A***, ***B***, Single virtual sections and corresponding models of representative tomograms of RIM-BP2 WT (***A***) and KO (***B***) AZs showing the AZ membrane (blue), SVs (green), and morphologically docked SVs (gray). Scale bars: 100 nm. ***C***, The AZ area estimated by the PSD, is significantly larger in the KO endbulb synapses; **p < 0.01, Student's *t* test. Each data point represents the AZ area of individual synapses. ***D***, The total number of SVs is not significantly different in mutant AZs; *p* > 0.05, Student's *t* test. Each data point represents the number of SVs per AZ of individual synapses. ***E***, SV number normalized to the AZ area is not significantly altered in KO endbulb AZs; *p* > 0.05, Wilcoxon rank test. Each data point represents the number of SVs normalized to the AZ area of individual synapses. ***F***, The number of morphologically docked SVs (0–2 nm) and SVs within 200 nm from the AZ membrane normalized to the AZ area divided into 20-nm bins. The number of docked as well as SVs within 2-20 and 40-60 nm is significantly lower in mutant AZs. The number of SVs in the other bins is not significantly different.; *p < 0.05, **p < 0.01, Wilcoxon rank test. Each data point represents the number of SVs in each bin normalized to the AZ area of individual AZs. ***G***, Frequency distribution of the number of morphologically docked SVs. ***H***, ***I***, Top views of representative tomogram models of RIM-BP2 WT (***H***) and KO (***I***) AZs with docked SVs. Scale bars: 100 nm. ***J***, Cumulative distribution of the lateral distances of morphologically docked SVs to the assumed center of the reconstructed AZ. ***K***, Frequency distribution of the SV number within 2–20 nm from the AZ membrane. (***L***) Mean SV diameter is not significantly altered in mutant synapses; *p* > 0.05, Student's *t* test. Each data point represents the mean diameter of SVs of individual synapses. Box and whisker plots present median, lower/upper quartiles, 10–90th percentiles. RIM-BP2 WT (*N* = 4; *n* = 26) in black and RIM-BP2 KO (*N* = 3; *n* = 28) in red (*N*: number of animals; *n*: number of AZs). For details about mean ± SEM, median, and statistics, see Table 8.

Next, we high-pressure froze aVCNs slices, acutely prepared as for physiology to best relate structure and function, for electron tomography analysis of SV organization at the endbulb AZ. Following freeze-substitution, embedding, sectioning and tomography, we rigorously analyzed and reconstructed AZ of RIM-BP2-deficient and WT endbulb synapses (Fig. 18). The AZ area, approximated from the extent of the postsynaptic density, was significantly larger in RIM-BP2-deficient endbulbs (Fig. 18*C*; Table 8, *p* < 0.01, Student's *t* test). The number (Fig. 18*D*) and density (Fig. 18*E*) of SVs per AZ were not altered. For a more comprehensive analysis we compared the SV distribution within 200 nm of the presynaptic AZ membrane (perpendicular to the presynaptic membrane into the cytosol of the presynaptic terminal) in 20-nm bins. Morphologically docked SVs (0- to 2-nm distance), analyzed separately, were significantly fewer in number at RIM-BP2 KO AZs. Significantly fewer SVs were also found for non-docked membrane proximal SVs (2–20 nm) and SVs within 40–60 nm from the AZ membrane (Fig. 18*F*, *p* < 0.01, Wilcoxon rank test; Table 8). We also analyzed top views of the AZs showing only the docked SVs in the models generated from the tomograms and quantified the percentage of AZs with zero to eight docked SVs. We found that 50% of the analyzed RIM-BP2 KO AZs showed zero docked SVs, while two docked SVs per AZ were most frequently encountered in WT (27% of the AZs; Fig. 18*G*). We further tested for effects of RIM-BP2 disruption on the lateral distribution of the docked SVs by setting a central point within the generated top views and quantifying the distances of all docked SVs from the center (we note that the AZ area captured in the tomograms might not necessarily allow proper definition of the AZ center). The docked SVs appeared to be further away from the center in RIM-BP2 KO synapses, possibly representing a broader distribution over the whole AZs area (Fig. 18*J*). To analyze the membrane proximal, non-docked SVs in more detail, we quantified the proportion of SVs in the 2- to 20-nm bin and observed a shift toward fewer SVs at mutant AZs. Whereas most of the WT (15%) AZs contained six SVs within 2–20 nm from the AZ membrane, most mutant AZs showed only two or five SVs within this distance (Fig. 18*K*). Lastly, by measuring the diameter of SVs, we found that they exhibited comparable sizes at RIM-BP2 KO and RIM-BP2 WT endbulb AZs (WT: 51.94 ± 0.55 nm; KO: 52.67 ± 0.54 nm). We conclude that RIM-BP2 contributes to normal SV docking and SV organization in close proximity to the membrane of the presynaptic AZ. These changes seem compatible with the lower number of superprimed SVs, impaired SV replenishment (Figs. 5, 7, 10) and the reduced release probability (Fig. 5).

**Figure 19.**
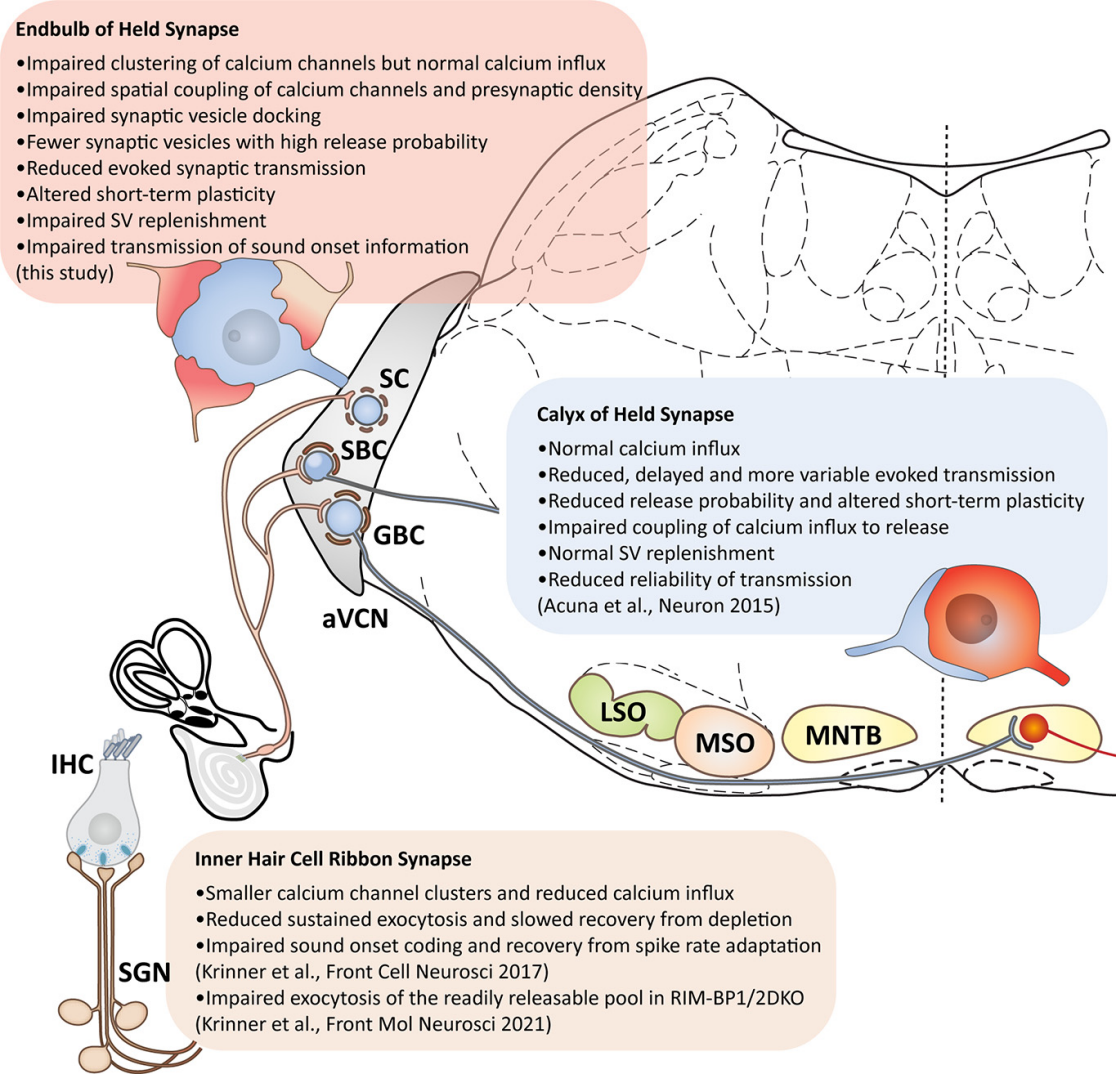
Compilation of findings on RIM-BP-deficient auditory synapses. RIM-BP2 is required for normal transmission at inner hair cell ribbon synapses as well as at endbulb and calyx of Held synapses. The roles of RIM-BP2 and RIM-BP1 have been addressed at the hair cell and the calyx of Held synapses, while our study focused on effects of deleting RIM-BP2 at the endbulb. The requirement for presynaptic tethering of Ca^2+^ channels seems most stringent at the hair cell synapse, but an altered localization of Ca^2+^ channels is manifested at all three synapses. Tight Ca^2+^ nanodomain coupling of Ca^2+^ channels and release ready SVs is maintained at hair cell synapses, while coupling is impaired at the calyx of Held, and this also seems to be case at the endbulb of Held. Both calyceal synapses show a major reduction in release probability, explanations of which include (1) impaired coupling of Ca^2+^ channels and release ready SVs and (2) impaired priming/docking of SVs (Fig. 18 of this study revealed a reduction of membrane proximal and docked SVs). This might relate to the interaction of RIM-BP to Munc13-1 described recently (Brockmann et al., 2020).

**Table 8.** Quantitative analysis of electron tomograms

Parameter		RIM-BP2 WT	RIM-BP2 KO	*p* value
AZ area (µm^2^)		0.0820 ± 0.0052 (0.0809)	0.1031 ± 0.0039 (0.0981)	**0.0022, T**
No. of SVs		49.81 ± 4.49 (46.50)	51.57 ± 4.63 (41.00)	0.79, T
Number of SVs per µm^2^ AZ	All SVs	662.3 ± 77.4 (543.1)	523.7 ± 53.4 (443.2)	0.14, W
	Docked SVs	27.52 ± 5.24 (18.58)	14.64 ± 4.77 (1.72)	**0.015, W**
	2–20 nm	109.1 ± 12.8 (98.4)	68.16 ± 9.22 (59.32)	**0.006, W**
	20–40 nm	58.59 ± 7.14 (45.26)	49.68 ± 5.40 (45.20)	0.61, W
	40–60 nm	83.25 ± 8.23 (82.21)	53.59 ± 5.36 (53.69)	**0.004, W**
	60–80 nm	79.81 ± 10.62 (64.31)	55.80 ± 6.26 (48.54)	0.09, W
	80–100 nm	70.43 ± 10.52 (54.67)	59.51 ± 7.92 (43.09)	0.39, W
	100–120 nm	61.41 ± 11.08 (43.38)	51.56 ± 9.16 (36.31)	0.51, W
	120–140 nm	50.51 ± 10.47 (41.23)	48.79 ± 7.41 (41.68)	0.64, W
	140–160 nm	55.12 ± 11.25 (42.14)	47.62 ± 6.21 (38.58)	0.82, W
	160–180 nm	37.59 ± 7.70 (25.56)	40.12 ± 8.33 (27.80)	0.92, W
	180–200 nm	29.68 ± 8.40 (15.05)	33.26 ± 4.97 (31.13)	0.20, W
Diameter (nm)	All SVs	50.99 ± 0.59 (51.68)	52.14 ± 0.68 (52.44)	0.73, T

Data are presented as mean ± SEM, and medians are shown in parentheses. Normal distribution was assessed with the Jarque–Bera test, and equality of variances was assessed with the *F* test in normally distributed data. Statistical significance of normally distributed data was determined by unpaired Student's *t* test (denoted as T), while that of the non-normally distributed data was determined by the Wilcoxon rank test (denoted as W). *p* < 0.05 set as threshold for statistical significance. Statistically significant differences shown in bold.

## Discussion

Priming of SVs, Ca_V_ function as well as the topography of Ca_V_ and the SV release sites at the AZ, co-determine the probability of SV release in response to the action potential invading the presynaptic terminal. Here, we probed the role of RIM-BP2, thought to serve as molecular linker between Ca_V_ and release sites, and alternatively in SV priming via Munc13, in synaptic transmission at the endbulb of Held synapse. Using superresolution immunofluorescence and immuno-electron microscopy we demonstrate that RIM-BP2 disruption alters the topography of Ca_V_2.1 channels at the AZs. Electron tomography revealed fewer docked and membrane-proximal SVs at the AZ. As a physiological corollary of these structural changes, we found a reduction in the amplitude of evoked EPSCs, reduced release probability, and slowed SV replenishment to the RRP. We postulate that RIM-BP2, likely via interaction with Munc13-1, promotes a superprimed (Taschenberger et al., 2016) or tightly docked (Neher and Brose, 2018) SV state. Moreover, RIM-BP2 organizes the topography of Ca_V_2.1 channels tightly coupling them to SV release sites.

Synaptic transmission at calyceal synapses of the lower auditory pathway shows impressive temporal fidelity. At the first central relay of the auditory pathway, co-incident transmission from endbulbs formed by ANF drives the postsynaptic BCs at hundreds of Hertz and with microsecond precision as required for time-critical neural computations such as in sound localization (Trussell, 1999; von Gersdorff and Borst, 2002). Such fidelity is enabled by synergistic adaptations on molecular, synaptic and network levels. In the mouse aVCN, BCs receive input from on average three to four endbulbs (Cao and Oertel, 2010; Butola et al., 2017) with ∼400 excitatory AZs (Nicol and Walmsley, 2002; Mendoza Schulz et al., 2014; Butola et al., 2017). Endbulbs feature a large RRP (Lin et al., 2011) and high release probability (estimates range from 0.2 to 0.7: Oleskevich and Walmsley, 2002; Wang and Manis, 2005; Chanda and Xu-Friedman, 2010; Mendoza Schulz et al., 2014; Butola et al., 2017) which enable massive synchronous parallel release for powerful excitation of BCs. RIM-BP2 deletion hampers this reliable and temporally precise transmission of auditory information: firing at sound onset was impaired both in rate and temporal precision (Fig. 8). We mainly attribute the reduced firing at sound onset (40% reduction) to the observed impairment of endbulb transmission (EPSC_1_ amplitude reduction: 36%; Fig. 1), as the deficit in sound onset coding in RIM-BP2 KO ANF was mild (∼10% reduction in peak onset firing rate; Krinner et al., 2017) and convergent ANF input to BCs is expected to alleviate consequences of impaired ANF coding for BC firing (Joris et al., 1994; Buran et al., 2010). These results from single-neuron recordings are in good agreement with previously reported auditory brainstem responses (Krinner et al., 2017), in which the amplitude reduction was lower for Wave I (∼30%) than for Wave III (∼70%). Waves I and III are attributed to the synchronized firing of ANFs and cochlear nucleus neurons, respectively (Melcher et al., 1996). Future studies, also employing analysis of gap detection or sound localization should evaluate the behavioral consequences of this temporal processing deficit.

Impaired transmission of sound onset information at RIM-BP2-deficient endbulbs is primarily rooted in a nearly halved initial release probability, which could be restored to WT levels on increasing Ca^2+^ influx (Fig. 10). We attribute the decreased release probability to (1) the altered topography of Ca_V_2.1 and SVs and (2) to the impaired SV docking at RIM-BP2-deficient endbulb AZs. Whole-cell patch-clamp recordings showed normal Ca^2+^ currents in RIM-BP2-deficient endbulbs (Fig. 4) and confocal imaging of Ca_V_2.1 immunofluorescence semi-quantitatively reported a normal Ca^2+^ channel complement of the AZs (Fig. 11). This suggests that unlike ribbon synapses (Krinner et al., 2017; Luo et al., 2017), RIM-BP2 is not strictly required for establishing a normal presynaptic Ca_V_ complement at the endbulb of Held synapse. However, both, FRIL and STED imaging of immunolabeled Ca_V_2.1 channels revealed an impaired clustering of Ca_V_2.1 at RIM-BP2-deficient endbulb AZs. The number and density of Ca_V_2.1 immunogold particles was reduced at the AZs (Table 6). Nonetheless, distribution of the Ca_V_2.1 immunogold particles was significantly different from random, likely reflecting the presynaptic Ca_V_ organization by other multidomain proteins of the AZ such as RIMs (Han et al., 2011; Kaeser et al., 2011; Jung et al., 2015) and CAST (Dong et al., 2018; Hagiwara et al., 2018). Immunofluorescence of Ca_V_2.1 as well as of Bsn (marking the presynaptic density) was less confined in space and immunofluorescent spots were more oval in shape in the absence of RIM-BP2 as compared to compact, round spots in the WT (Fig. 12, 13). The nearest neighboring immunofluorescence spot was further away both for Ca_V_2.1 and Bsn, implying that both Ca_V_2.1 and Bsn clusters were further apart from their respective Ca_V_2.1 and Bsn clusters in the mutant synapses. In addition, when checking the Ca_V_2.1 to Bsn distance, we found that the Ca_V_2.1 clusters were further apart from the Bsn clusters in the mutant (Fig. 14), implying an increased distance between the Ca_V_2.1 and SV release machinery marked by Bsn. In summary, our data indicate that RIM-BP2 contributes to orchestrating Ca_V_2.1 channels at the AZ. Based on our data and in line with previous studies on other synapses (Liu et al., 2011; Acuna et al., 2015; Brockmann et al., 2020; Petzoldt et al., 2020), we hypothesize that RIM-BP2 via its interaction with Ca_V_2.1 and AZ proteins, contributes to tight coupling of Ca^2+^ channels and vesicular release sites also at the endbulb of Held synapse. Future experimental and theoretical studies will be needed to further test this hypothesis.

Recently, an alternative interaction of RIM-BP2 with the C2B domain of Munc13-1 has been reported by which RIM-BP promotes release probability via SV docking/priming (Brockmann et al., 2020). The Munc13-1 C2B domain carrying the KW mutation (Shin et al., 2010), showed the highest affinity for RIM-BP2 binding. Of note, we did not observe a reduction in Munc13-1 or in any other major AZ protein in our semi-quantitative analysis of AZs in RIM-BP2-deficient endbulbs of Held (Fig. 9). This provides confidence in attributing functional and morphologic alterations to the lack of RIM-BP2 function rather than to quantitative changes in other AZ proteins. Our functional and electron tomographic analysis of SV pool organization at RIM-BP2-deficient endbulbs now provides evidence for a role of RIM-BP in SV priming, likely via its interaction with Munc13-1. Rigorous electron tomography analysis of docking showed that the numbers of morphologically docked SVs (defined in a distance from 0 to 2 nm to the AZ membrane) were nearly halved at RIM-BP2-deficient AZs, which might represent a reduced tight docking (Neher and Brose, 2018). We speculate this to indicate that Munc13-1-mediated docking that generates primed SVs (Siksou et al., 2009; Imig et al., 2014) partially depends on the interaction with RIM-BP2. The reduction of both docked SVs and those at a distance of 2–20 nm (membrane proximal SVs) from the plasma membrane might then suggest that the upstream loose-docking is rate limiting or less stable in the absence of RIM-BP2. An alternative explanation for retarded recovery from depression might be that RIM-BP2 facilitates release site clearance (Neher and Sakaba, 2008).

However, our analysis of pool dynamics in regular [Ca^2+^]_e_ indicated an unaltered RRP size despite the fact that the numbers of docked and membrane-proximal SVs were nearly halved. Interestingly, we found a trend toward a smaller RRP in RIM-BP2-deficient endbulbs when restoring release probability by enhanced Ca^2+^ influx. Despite this partial restoration, the first evoked EPSC amplitude in the absence of RIM-BP2 was still only 50% of that in the WT synapses (Fig. 10; Table 4). This persisting difference alludes to a deficit in superprimed (Fig. 5; Table 2) or tightly docked SVs (Fig. 18; Table 8). It is tempting to speculate that the impaired SV docking is uncovered under conditions that occlude the effect of altered spatial coupling of Ca^2+^ channel-release site on release probability. Our morphologic analysis of Ca^2+^ channel-release site coupling is hampered by (1) the low number of docked SVs in electron tomography and (2) the lack of information on the Ca_V_ position in electron tomography and of SV docking in FRIL electron micrographs. Nonetheless, there was a trend for docked SVs to be further away from the estimated AZ center, possibly reflecting a more random SV topography because of lack of RIM-BP2-mediated interaction with Ca_V_s. In addition, or alternatively, the RRP estimated by SMN/EQ analyses of responses to train stimulation might contain SVs that undergo tethering, docking and fusion during the train. Recent work by Pofantis et al. (2021), showed that deleting the presynaptic protein Mover specifically affects the high P_r_ component of the RRP at the Calyx of Held. eEPSC trains were analyzed with non-negative matrix factorization to reveal components representing subpools of SVs with different contributions to transmitter release during the train. Future studies that tackle how such subpools deplete and how they recover from depletion in synapses lacking both RIM-BP2 and Mover will help to further dissect the contributions of RIM-BP2 to SV superpriming and Ca_V_ clustering as determinants of P_r_.

Exciting topics for future studies include i) the relative contributions of altered Ca^2+^ channel-release site coupling and impaired SV docking to the reduced release probability, ii) a potential contribution of release site clearance to the observed deficit in SV docking, and iii) the molecular mechanisms and structure-function relationship of SV replenishment. Our morphologic and functional experiments indicate that RIM-BP2 takes a role in tethering SVs near the plasma membrane en route to docking. Functionally, the fast, Ca^2+^-dependent component of recovery from depression because of trains stimulation was hampered. This could reflect a lower local [Ca^2+^] because of mislocalization of Ca_V_2.1 channels. Indeed, a key role of Ca^2+^ in regulating SV replenishment at calyceal synapses has been reported in multiple studies (Wang and Kaczmarek, 1998; Hosoi et al., 2007). Interestingly, enhancing Ca^2+^ influx by elevated [Ca^2+^]_e_ uncovered a reduced SV replenishment during train stimulation, which would seem to reflect a Ca^2+^ independent limitation e.g., of SV priming. Future studies on calyceal synapses of mice carrying mutations that target Ca^2+^-dependent effects on Munc13-1 function and/or the RIM-BP2-Munc13-1 interactions by combined functional and ultrastructural analyses will be required to elucidate this intricate process. To best relate AZ structure and function, future studies will ideally employ optogenetic or electric field stimulation followed by high-pressure freezing and EM tomography (Watanabe et al., 2013; Imig et al., 2020).
